# Total transcriptome analysis of *Candida auris* planktonic cells exposed to tyrosol

**DOI:** 10.1186/s13568-023-01586-z

**Published:** 2023-08-02

**Authors:** Noémi Balla, Ágnes Jakab, Fruzsina Kovács, Ágota Ragyák, Zoltán Tóth, Dávid Balázsi, Lajos Forgács, Aliz Bozó, Farah Al Refai, Andrew M Borman, László Majoros, Renátó Kovács

**Affiliations:** 1 Department of Medical Microbiology, Faculty of Medicine, University of Debrecen, Nagyerdei krt. 98, Debrecen, 4032 Hungary; 2 Doctoral School of Pharmaceutical Sciences, University of Debrecen, Debrecen, 4032 Hungary; 3 Department of Molecular Biotechnology and Microbiology, Institute of Biotechnology, Faculty of Science and Technology, University of Debrecen, Debrecen, Hungary; 4 Department of Inorganic and Analytical Chemistry, Agilent Atomic Spectroscopy Partner Laboratory, University of Debrecen, Debrecen, Hungary; 5 UK National Mycology Reference Laboratory, Public Health England, Science Quarter, Southmead Hospital, Bristol, BS10 5NB UK; 6 Medical Research Council Centre for Medical Mycology (MRCCMM), University of Exeter, Exeter, EX4 4QD UK

**Keywords:** *Candida auris*, Tyrosol, Oxidative stress, Quorum-sensing, Iron, Metal, Treatment

## Abstract

Tyrosol, a secondary metabolite of *Candida* species, regulates fungal morphogenesis, and its application may represent a novel innovative therapy against emerging multi-resistant fungal superbug such as *Candida auris*. In the current study, the effects of tyrosol on growth, redox homeostasis, intracellular microelement contents and activities of virulence-related enzymes released by *C. auris* were examined. To gain further information about the effect of tyrosol exposure, we revealed gene transcriptional changes using total transcriptome sequencing (RNA-Seq). At a concentration of 15 mM, tyrosol significantly decrease the growth of fungal cells within 2 h of its addition (5.6 × 10^7^±1.2 × 10^7^ and 2.5 × 10^7^±0.6 × 10^7^ colony forming unit/mL for control and tyrosol-treated cells, respectively). Furthermore, it enhanced the release of reactive oxygen species as confirmed by a dichlorofluorescein (DCF) assay (7.3 ± 1.8 [nmol DCF (OD_640_)^−1^] versus 16.8 ± 3.9 [nmol DCF (OD_640_)^−1^]), which was coincided with elevated superoxide dismutase, catalase and glutathione peroxidase activities. Tyrosol exerted in a 37%, 25%, 34% and 55% decrease in intracellular manganese, iron, zinc and copper contents, respectively, compared to control cells. The tyrosol treatment led to a 142 and 108 differentially transcripted genes with at least a 1.5-fold increase or decrease in transcription, respectively. Genes related to iron and fatty acid metabolism as well as nucleic acid synthesis were down-regulated, whereas those related to the antioxidative defence, adhesion and oxoacid metabolic processes were up-regulated. This study shows that tyrosol significantly influences growth, intracellular physiological processes and gene transcription in *C. auris*, which could highly support the development of novel treatment approaches against this important pathogen.

**Supplementary Information:**

The online version contains supplementary material available at 10.1186/s13568-023-01586-z.

## Key Points

– Tyrosol treatment showed 615 differentially expressed genes in *C. auris*.

– Tyrosol generates oxidative stress in *C. auris*.

– Tyrosol decreased the iron, zinc, manganese and copper content in *C. auris*.

**Supplementary Information:**

The online version contains supplementary material available at 10.1186/s13568-023-01586-z.

## Introduction

The World Health Organization reported the first fungal priority pathogen list in the second half of 2022, where *Candida auris* was assigned to the critical priority group (WHO 2022). Once this species becomes established in a health-care facility, it is almost impossible to eliminate. There is a solid consensus among healthcare workers that the alarming spread of multi- and pan-resistant *C. auris* isolates highlights the need for the development of novel antifungal drugs and/or innovative therapeutic solutions against this fungal superbug (McCarthy et al. 2017; CDC 2019; Chaves et al. 2021).

Molecules that interfere with quorum-sensing are promising future opportunities for the management of different *Candida* infections (Nett et al. 2014; Kovács et al. 2020; Costa et al. 2021). In recent years, the application of fungus derived quorum-sensing molecules (farnesol or tyrosol) alone or combined with traditional antifungal agents against various fungal pathogens has received considerable research interest (Shanmughapriya et al. 2014; Cordeiro et al. 2015; Monteiro et al. 2017; Kovács et al. 2017; Nagy et al. 2020/a). Tyrosol, (2-(4-hydroxyphenyl)-ethanol, is a phenolic compound derived from phenethyl alcohol, which has a pivotal role in regulation of *Candida* morphogenesis and biofilm formation especially in *C. albicans* (Chen et al. 2004). At a concentration of 20 µM, tyrosol induces germ tube development and hyphal formation in the initial stages of biofilm development, particularly in the first 6 h (Alem et al. 2006; Chen et al. 2014). Notably, *Candida albicans*-derived findings cannot be extrapolated to certain non-*albicans* species. Jakab et al. (2019) reported that the presence of tyrosol increase the oxidative stress and the transcription of genes encoding efflux pumps, whereas it inhibited ribosome biogenesis and growth as well as several virulence associated genes in case of *Candida parapsilosis* (Jakab et al. 2019). Moreover, metabolic profile was altered toward ethanol fermentation and glycolysis, whereas initial adherence was not significantly affected compared to *C. albicans* (Jakab et al. 2019). In other studies, tyrosol exhibited a potent antifungal and anti-virulence activity both in vitro and in vivo, especially at supraphysiological concentrations, against different fungal species including *C. albicans*, *Candida tropicalis* and *C. parapsilosis* (Shanmughapriya et al. 2014; Cordeiro et al. 2015; Kovács et al. 2017; Jakab et al. 2019; Nagy et al. 2020/a). However, these studies focused mainly on the phenotypic responses, often without a detailed molecular biology-based explanation for the observed effect. Previous studies revealed that *C. auris* showed different physiological responses to exposure to various secondary metabolites compared to *C. albicans* or other non-albicans species (Chauhan et al. 2020; Begum et al. 2022). For instance, it is more susceptible to farnesol treatment in vitro and in vivo compared to *C. albicans*, which can be exploited for the development of new promising antifungal treatment opportunities (Nagy et al. 2020/b).

In the current paper, we report the impact of tyrosol treatment on the growth and total gene transcription profile of *C. auris*. Our results provide, for the first time, a tool for analysing the global pattern of gene transcription of this species, which may help to explain the physiological role of this quorum-sensing molecule and the molecular explanation of possible antifungal effects in *C. auris*.

## Materials and methods

### Fungal isolate and culture condition

Whole genome-sequenced *C. auris* isolate 12 (NCPF 8973), received from the National Mycology Reference Laboratory, Bristol, United Kingdom, was used (accession no.: JANPVY000000000) (Balla et al. 2022). The strain is the member of the South Asian/Indian clade and showed a non-aggregating phenotype (Borman et al. 2017). It was stored as frozen stock containing 20% glycerol at − 80 ºC and subcultured on standard yeast peptone dextrose (YPD) agar (2% mycological peptone, 1% yeast extract, 2% glucose with 2% agar, at pH 5.6). Tyrosol (2-(4-hydroxyphenyl)-ethanol; Merck, Budapest, Hungary) was diluted as a 0.1 M stock solution in standard YPD for all performed experiments.

### Growth-related experiments

The pre-cultures were routinely grown in standard YPD broth at 30ºC with a 2.3-Hz shaking frequency for 18 h and subsequently diluted to a value of 0.1 (at OD_640_) (~ 2.5 ± 0.5 × 10^6^ colony forming unit (CFU)/mL) in 20 mL of YPD, followed by incubation at 37ºC with a 2.3-Hz shaking frequency. After 4 h-long incubation period, tyrosol was pipetted at a final concentration of 15 mM to the *C. auris* cultures in YPD. Afterwards, growth was followed continuously at 1-hour intervals by the measuring of cell density and determination of living cell number via measuring the absorbance (at 640 nm) and quantitative culturing, respectively. The applied tyrosol concentration is safe and can be tolerated by different human cell lines as examined in our previous study (Jakab et al. 2019). At sampling timepoints, tyrosol induced morphological changes were examined using phase-contrast microscopy. Statistical analysis of growth-based data was carried out by the paired Student’s *t*-test using the GraphPad Prism 6.05 software (GraphPad Software, LLC, Boston, USA). The statistical differences between tyrosol-treated and control values were considered significant if the *p* value was lower than 0.05.

### Determination of aspartic proteinase, esterase and extracellular phospholipase activites

To determine the extracellular phospholipase activity of tyrosol-exposed (15 mM) and unexposed *C. auris* cells, egg yolk medium [5.85% (w/v) NaCl, 0.05% (w/v) CaCl_2_ and 10% (w/v) sterile egg yolk (Merck, Budapest, Hungary) in solid YPD medium] was used; while esterase activity was assessed on Tween-20 medium [1% peptone, 0.5% NaCl, 0.01% CaCl_2_ × 2H_2_O, 1% (v/v) Tween-20 (Merck, Budapest, Hungary), 2% agar]. The activity of aspartic proteinase was examined on solid medium supplemented with bovine serum albumin (Merck, Budapest, Hungary) [0.02% (w/v) MgSO_4_ × 7H_2_O, 0.25% (w/v) K_2_HPO_4_, 0.5% (w/v) NaCl, 0.1% (w/v) yeast extract, 2% (w/v) glucose and 0.25% (w/v) bovine serum albumin (Merck, Budapest, Hungary) agar medium]. For the assays, 5 µL of 1 × 10^7^ cells/mL suspensions were inoculated onto the surface of agar plates as described previously, and diameter of colonies and precipitation zones on egg yolk and Tween-20 plates or clear zones on bovine serum albumin media were measured following 7 days-long incubation period at 37 °C (Nagy et al. 2020/b). Enzyme activities were measured from three independent biological repeats and are presented as means ± standard deviations. Afterwards, activities were analyzed by paired Student’s *t*-test, using the GraphPad Prism 6.05 software (GraphPad Software, LLC, Boston, USA). The statistical differences between tyrosol-treated and control values were considered significant if the *p* value was lower than 0.05.

### Reactive species production, reduced glutathione, oxidized glutathione contents and antioxidant enzyme activities

For the evaluation of reactive species release and antioxidant enzyme activities, pre-cultures were diluted to a value of 0.1 (OD_640_) in YPD broth, and then were grown for 4 hours at 37ºC. Afterwards, YPD medium was supplemented with a final concentration of 15 mM tyrosol, and *C. auris* cells were collected over a 2-hour-long tyrosol treatment by centrifugation (5 min, RCF = 4,000 g, at 4°C). Quantitative analysis of reactive species was performed with and without tyrosol by a reaction that converts 2’,7’-dichlorofluorescin diacetate to 2’,7’-dichlorofluorescein (DCF) (Merck, Budapest, Hungary). The produced DCF is directly proportional to the quantity of reactive species. Reduced and oxidised glutathione (GSH and GSSG) contents of cells were determined with the 5,5′-dithiobis-(2-nitrobenzoic acid) (DTNB, GR assay) according to Jakab et al. (2015). Oxidative stress parameters (superoxide dismutase (SOD), catalase, glutathione reductase (GR), glutathione peroxidase (GPx) were determined as published previously by Jakab et al. (2015). Reactive species and enzyme activities were measured from three independent biological experiments and presented as means ± standard deviation. Statistical comparison of reactive species and enzyme production data was performed by the paired Student’s *t*-test, using the GraphPad Prism 6.05 software (GraphPad Software, LLC, Boston, USA). The differences between tyrosol-exposed and unexposed values were considered significant if the *p* value was < 0.05.

### Measurement of microelement content of *Candida auris* cells

The *C. auris* pre-cultures were cultured, and tyrosol exposure was performed as detailed above. Fungal cells were collected by centrifugation (5 min, RCF = 4,000 g, at 4 °C) after 2 h-long of incubation with tyrosol. Reduction in *C. auris* dry cell mass (DCM) was assessed after freeze-drying (Jakab et al. 2021). The intracellular microelement profiles of obtained DCM were measured by inductively coupled plasma-optical emission spectrometry (ICP-OES; 5110 Agilent Technologies, Santa Clara, USA) following atmospheric wet digestion in 3 mL of 65% (M/M) HNO_3_ and 1 mL of 30% (M/M) H_2_O_2_ in glass beakers. The iron, zinc, manganese and copper contents of three independent samples were calculated and expressed in DCM units (mg/kg) as described previously by Jakab et al. (2021). The metal biomass contents were determined in three independent biological replicates and means with standard deviation values were calculated and presented. The statistical significance of changes was determined by Student’s *t*-test. The differences between tyrosol-exposed and unexposed values were considered significant if the *p* value was < 0.05.

### Transcriptional profiling

Total RNA content was isolated from unexposed control *C. auris* cells and 15 mM tyrosol-treated fungal cell cultures from three independent biological experiments. Freeze-dried cell mass was processed using TRISOL (Thermo Fisher Scientific, Invitrogen, Waltham, USA) reagent as published by Chomczynski (1993).

Illumina NextSeq sequencing platform was used to carry out high-throughput mRNA sequencing and to obtain global transcriptome data. To evaluate the total RNA sample quality, Agilent BioAnalyzer was applied using the Eukaryotic Total RNA Nano Kit (Agilent technologies, Inc., Santa Clara, CA, USA) as described in manufacturer’s protocol. Library preparation was performed from samples with an RNA integrity number (RIN) higher than 7. NEBNext Ultra II RNA sample preparation kit (New England Biolabs, Ipswich, USA) was used to prepare the RNA-Seq libraries from total RNA content based on the manufacturer’s instructions. Both sequencing and the library preparation were performed by the Genomic Medicine and Bioinformatics Core Facility of University of Debrecen, Hungary. The single-read sequencing reads (75-bp-long) were generated on an Illumina NextSeq500 instrument. About 18 to 22 million reads per sample were generated and raw reads were aligned to the reference genome (genome, https://fungi.ensembl.org/_candida_auris_gca_002759435/Info/Index;

features,http://www.candidagenome.org/download/gff/C_auris_B8441/archive/C_auris_B8441_version_s01-m01 r11_features_with_chromosome_sequences.gff.gz), and aligned reads varied between 90 and 95% in samples. To obtain normalized gene transcription values, the DESeq algorithm (StrandNGS software) was applied. Gene transcription differences between treated and untreated groups were compared by a moderated *t-*test; the Benjamini-Hochberg false discovery rate was used for multiple-testing correction, and a corrected *p* < 0.05 was considered to represent a significant difference. Exclusively genes up- or down-regulated significantly with more than 1.5-fold change (FC) values were taken into further detailed consideration. The FC ratios were calculated from the normalized gene transcription values.

### Reverse transcriptase-quantitative polymerase chain reaction (RT-qPCR) assays

Six up-regulated and five down-regulated genes, as well as five genes without significant transcriptional changes, were selected for further RT-qPCR analysis to confirm the obtained RNA-Seq results. Xceed qPCR SG 1-step 2x Mix Lo-ROX kit (Institute of Applied Biotechnologies, Prague, Czech Republic) were performed according to the manufacturer’s instructions, using 500 ng of DNase-treated (Merck, Budapest, Hungary) total RNA/reaction. OligoExplorer (version 1.1.) and Oligo Analyser (version 1.0.2) were used to design primer pairs (Supplemental Table S1), purchased from Integrated DNA Technologies. Three independent biological replicates were performed with each sample in a LightCycler® 480 II Real-Time PCR instrument (Roche, Basel, Switzerland). The relative transcription level (ΔΔCP) was calculated using the difference between the crossing points of the reference gene (actin; *B9J08_000486*) and the target gene within a sample (ΔCP) (Jakab et al. 2019, 2022). The ΔΔCP values are presented as means with standard deviations, calculated from three biologically independent measurements, and were compared using Student’s *t*-test.

### Gene set enrichment analysis

Significant shared GO terms were determined with Candida Genome Database Gene Ontology Term Finder (http://www.candidagenome.org/cgi-bin/GO/goTermFinder). Only records with a *p* value < 0.05 were taken into further consideration in detailed gene set enrichment analysis. Enrichment of selected gene groups in the functionally related up-regulated and down-regulated gene sets was also studied with Fisher’s exact test (*p* < 0.05, www.R-project.org/). The following gene categories were analysed:

i. “Virulence-associated genes” are genes collected by Jakab et al. (2021);
ii. “Carbohydrate, ergosterol and fatty acid metabolic pathway-related genes”. based on the pathway databases (http://pathway.candidagenome.org/);
iii. “Genes involved in response to oxidative stress”.
iv. “Iron, zinc, manganese and copper metabolism-associated genes” are genes collected by Jakab et al. (2021).

## Results

### Effects of tyrosol on growth, morphological changes, extracellular phospholipase, proteinase and lipase production

**Fig. 1:**
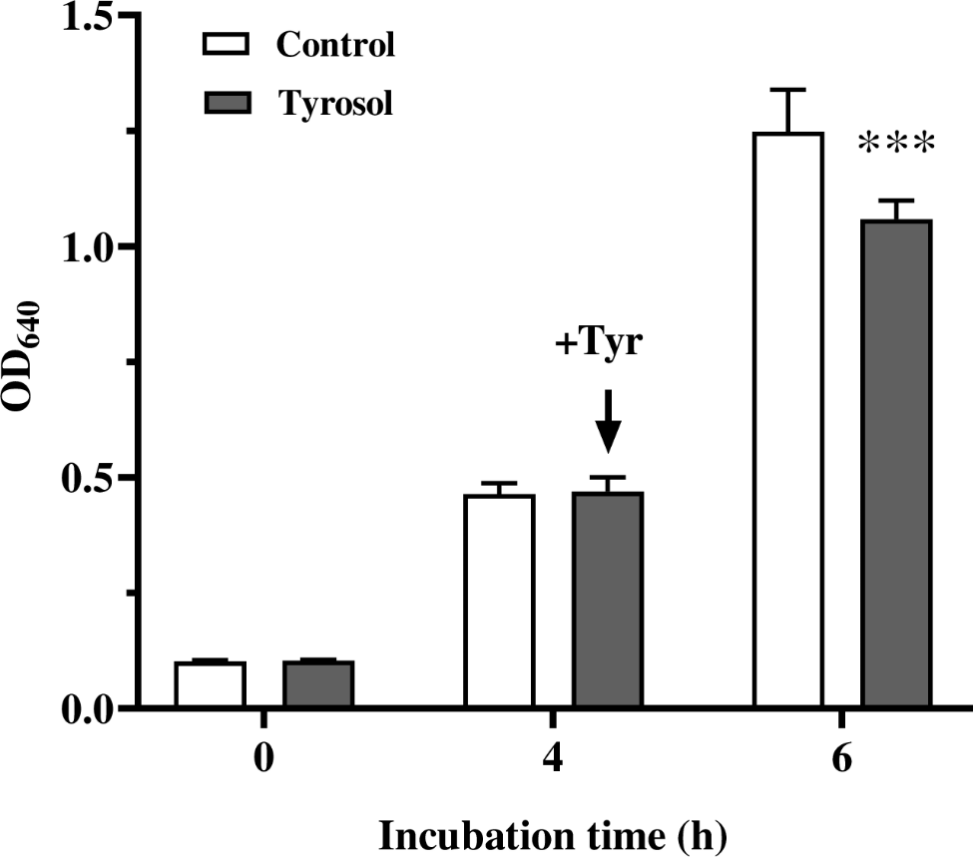
Effect of tyrosol on the growth of *Candida auris*. Growth of *C. auris* was followed in YPD medium by measuring of absorbance (OD_640_). Tyrosol was added at 4 h incubation time in 15 mM final concentration. Figures represent mean ± standard deviation values calculated from three independent biological replicates. Asterix symbol represents significant difference between control and tyrosol treated cultures calculated by paired Student’s *t*-test

Growth rate of *C. auris* was followed after 15 mM tyrosol treatment in YPD medium (Fig. 1). Growth was significantly inhibited following 2 h of tyrosol exposure as measured using both CFU changes (5.6 × 10^7^ ± 1.2 × 10^7^ and 2.5 × 10^7^ ± 0.6 × 10^7^ CFU/mL for control and tyrosol-exposed cells, respectively) (*p* < 0.001) and changes in absorbance values of 1.25 ± 0.09 and 1.06 ± 0.04 [OD_640_]) (*p* < 0.001) (Fig. 1). The morphology of *C. auris* cells was not significantly altered by exposure to tyrosol. The ratio of yeast and pseudohyphae did not differ significantly between tyrosol-exposed cells (88% ± 4% and 12% ± 2% for yeast and pseudohyphae, respectively) and control cells (84% ± 4% and 16% ± 3% for yeast and pseudohyphae, respectively) with a 2-hour-long exposure time (*p* > 0.05). For the virulence factors examined, the extracellular proteinase activity did not differ significantly in tyrosol-treated and control cells (precipitation zone [Pz] values were 0.82 ± 0.04 and 0.79 ± 0.04 for unexposed and tyrosol-exposed cells, respectively; *p* > 0.05). Similar pattern was observed in phospholipase activity for unexposed control and tyrosol-exposed cells (the Pz values were 0.88 ± 0.01 and 0.85 ± 0.03 for control and tyrosol-exposed cells, respectively; *p* > 0.05). Additionally, tyrosol treatment did not significantly affect the activity of lipase of fungal cells. The calculated Pz values were 0.65 ± 0.04 and 0.61 ± 0.07 for *C. auris* unexposed control and tyrosol-exposed cells, respectively (*p* > 0.05).

### **Tyrosol exposure generates oxidative stress in*Candida auris***

Tyrosol treatment significantly increase the reactive oxygen species production compared to untreated *Candida* cells (16.8 ± 3.9 [nmol DCF (OD_640_)^−1^] versus 7.3 ± 1.8 [nmol DCF (OD_640_)^−1^]) (*p* < 0.05) (Table 1). The higher reactive species level observed was in line with elevated superoxide dismutase, catalase and glutathione peroxidase activities and an increased level of GSH (*p* < 0.05) in tyrosol-exposed cells (Table 1). In contrast, the measured glutathione reductase values and oxidised glutathione contents were not differ significantly (*p* > 0.05) in tyrosol-treated and untreated fungal cells (Table 1).

**Table 1:** Tyrosol-induced oxidative stress response in *Candida auris*

Oxidative stress related parameter	Untreated cultures	Tyrosol-treated cultures
2’,7’–dichlorofluorescein [nmol DCF (OD_640_)^−1^]	7.1 ± 1.9	16.8 ± 3.9^*^
Superoxide dismutase [munit (mg protein)^−1^]	54.3 ± 6.1	78.1 ± 14.8 ^*^
Catalase [kat (kg protein)^−1^]	0.6 ± 0.2	1.2 ± 0.35 ^*^
Glutathione reductase [mkat (kg protein)^−1^]	2.2 ± 0.5	2.8 ± 0.3
Glutathione peroxidase [mkat (kg protein)^−1^]	0.2 ± 0.02	0.29 ± 0.06 ^*^
Reduced glutathione [nmol (OD_640_)^− 1^]	49.8 ± 5.4	65.1 ± 4.0 ^*^
Oxidized glutathione [nmol (OD_640_)^− 1^]	0.2 ± 0.04	0.26 ± 0.04

Mean ± standard deviation values calculated from three independent biological replicates are presented

^*, ***^ Significant differences at *p* < 0.05 and 0.001, respectively, as calculated by the paired Student’s *t*-test compared to untreated control and tyrosol-treated cultures

### Tyrosol exposure significantly influences the intracellular level of microelements in *Candida auris* cells

Tyrosol exposure led to a 25%, 37%, 34% and 55% decrease in intracellular iron, manganese, zinc and copper content, respectively, compared to untreated control cells (155.7 ± 17.9 mg/kg versus 114.3 ± 16.2 mg/kg, 69.3 ± 4.2 mg/kg versus 42.6 ± 1.14 mg/kg, 735.4 ± 18.5 mg/kg versus 516.5 ± 22.1 mg/kg, and 259.9 ± 16.1 mg/kg versus 123.5 ± 9.8 mg/kg for iron, manganese, zinc and copper, respectively) (*p* < 0.05 to 0.001), as presented in Table 2 (*p* < 0.001).

**Table 2:** Tyrosol induced changes in the metal content in Candida *auris*

Metal contents/ Treatment	Mean value ± SD^a^				
	Dry cell mass (g/l)	Fe (mg/kg)	Mn (mg/kg)	Zn (mg/kg)	Cu (mg/kg)
Untreated cultures	0.32 ± 0.02	155.7 ± 17.9	69.3 ± 4.2	735.4 ± 18.5	259.9 ± 16.1
Tyrosol-treated cultures	0.26 ± 0.03^**^	114.3 ± 16.2 ^*^	42.6 ± 1.14 ^**^	516.5 ± 22.1 ^**^	123.5 ± 9.8 ^**^

^a^ Mean values **±** standard deviations (SD) calculated from three independent biological replicates are presented

* and ** indicate significant differences at *p* values of < 0.05 and 0.01, calculated by two-way ANOVA, comparing untreated control and tyrosol-treated cultures

### Transcriptional profiling and RNA-Seq data validation

**Fig. 2:**
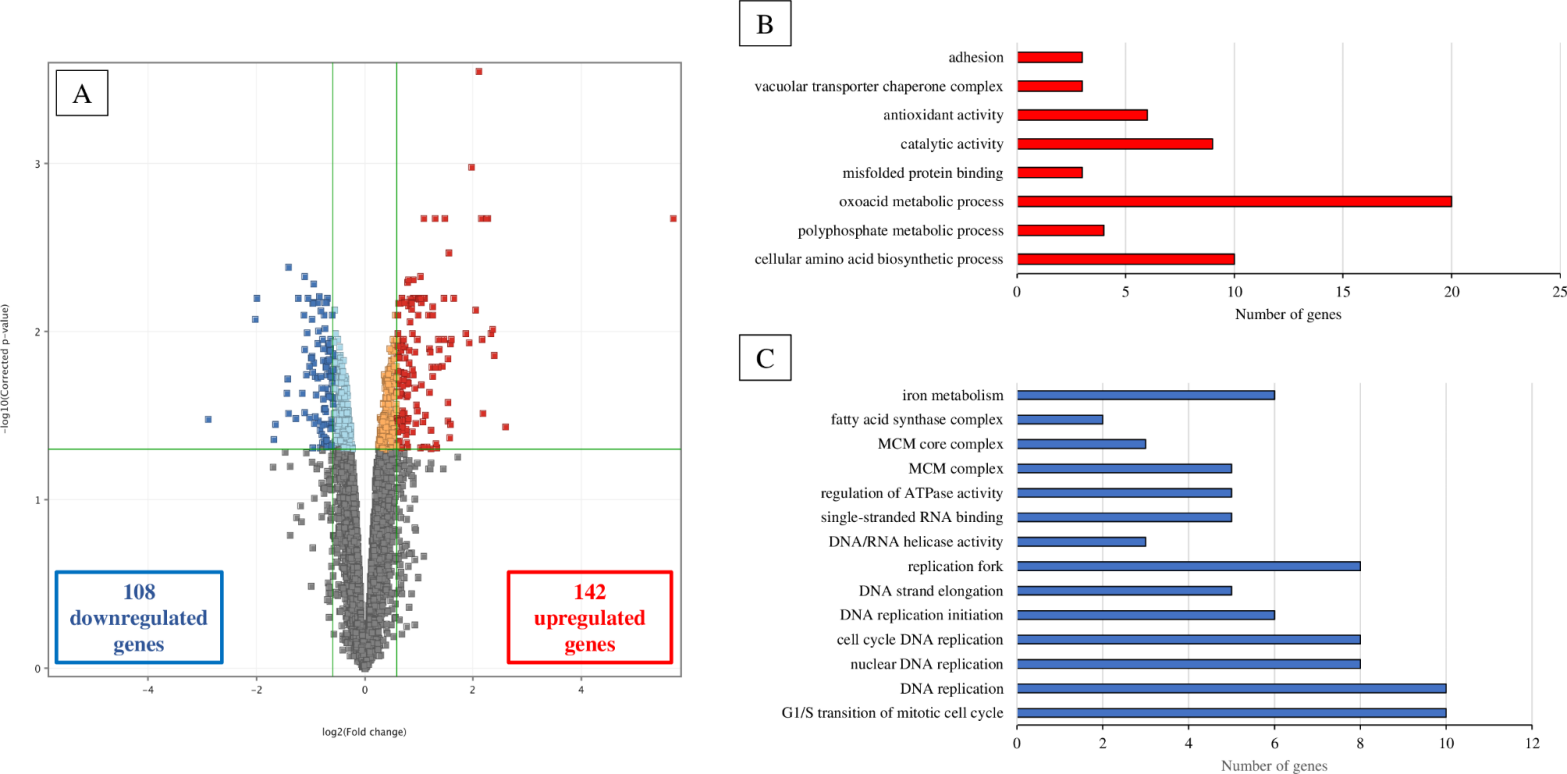
Overview of transcriptional changes induced by tyrosol in *C. auris*. Up-regulated (red) and down-regulated (blue) genes were defined as differentially expressed genes (corrected *p* value of < 0.05), with more than a 1.5-fold increase or decrease in their transcription (tyrosol-treated versus untreated) (A). Summary of gene enrichment analyses and the number of genes affected by *C. auris* exposure to tyrosol (B–C). The enrichment of up-regulated (B) and down-regulated (C) gene groups was identified using the Candida Genome Database Gene Ontology Term Finder (http://www.candidagenome.org/cgi-bin/GO/goTermFinder) or was tested by Fisher’s exact test. The data sets for the gene groups are available in Supplemental tables S2 and S3 in the supplemental material

**Fig. 3:**
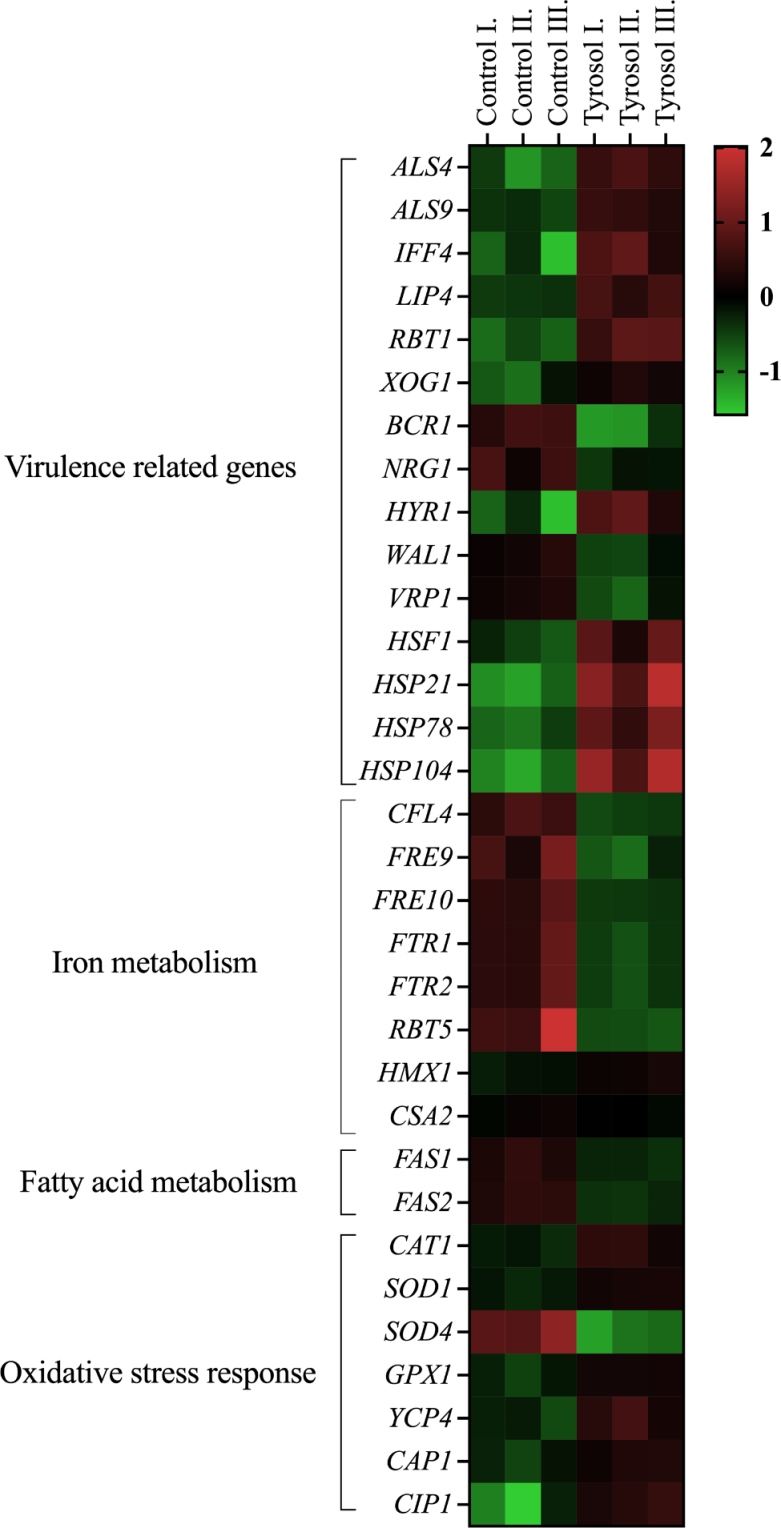
The effects of tyrosol on the expression of selected genes of *C. auris*. The heat map demonstrates the transcription profiles of representative genes according to the colour scale that indicates gene transcription changes. The data sets for the gene groups are available in supplemental tables S2, S3 and S4

The tyrosol-exposed *C. auris* gene transcription profile revealed 615 differentially transcribed genes (*p* < 0.05) compared to untreated control (Figs. 2 and 3, Supplemental Fig. S1). The qRT-PCR data of 16 genes selected exhibited high positive correlation with the presented RNA-Seq data (Fig. 3). The results of the gene set enrichment analysis of the 142 up-regulated (log_2_(FC) > 0.585) and 108 down-regulated (log_2_(FC) < -0.585) genes are presented in Supplemental Tables S2, S3 and S4; and further detailed below.

### Evaluation of tyrosol responsive genes

#### Virulence-associated genes

Genes associated with adhesion (*ALS4*, *ALS9* and *IFF4)*, lipase secretion (*LIP4*), biofilm maturation (*RBT1* and *XOG1*), morphogenesis (*HYR1*) and stress response (*HSF1*, *HSP21*, *HSP78* and *HSP104*) were enriched in the up-regulated gene set. In addition, tyrosol exposure induced a decrease in the transcription of *BCR1* (biofilm maturation), *NRG1* (biofilm dispersion) *WAL1* and *VRP1* (morphological transition) genes. In addition, up-regulation of *LIP4*, *RBT1* and *HSP21* and down-regulation of *NRG1* under tyrosol treatment was further confirmed using RT-qPCR (Figs. 2 and 3; Supplemental tables S2, S3 and S4).

#### Metabolic pathway-associated genes

Tyrosol treatment resulted in a decreased transcription of several genes involved in reductive iron uptake (*CFL4*, *FRE9*, *FRE10*, *FTR1* and *FTR2*), haemoglobin use (*RBT5*, *HMX1* and *CSA2*) and fatty acid biosynthesis (*FAS1* and *FAS2*) but not carbohydrate and ergosterol metabolisms. The down-regulation of *FAS1*, *FAS2* and *FRE1* was additionally confirmed by RT-qPCR (Fig. 3). In addition, tyrosol exposure decreased the transcription of genes related to DNA replication (10 genes) and MCM complex (5 genes) (*p* < 0.05) (Figs. 2 and 3; Supplemental Tables S2, S3 and S4).

#### Oxidative stress-associated genes

Based on performed Fisher’s exact test, genes belonging to the “antioxidative defence-related gene” were enriched in the tyrosol-responsive up-regulated gene group. Tyrosol exposure increased the transcription of genes encoding catalase (*CAT1*), superoxide dismutase (*SOD1*), glutathione peroxidase (*GPX1*) as well as flavodoxin-like protein (*YCP4*) and the *CAP1* and *CIP1* genes, which encode the major regulator in oxidative stress defence and an environmental stress-induced protein. In addition, tyrosol exposure caused a decrease in the transcription of the *SOD4* gene. Up-regulation of *CAT1* and *CIP1* as well as down-regulation of *SOD4* following tyrosol exposure was confirmed by RT-qPCR data (Figs. 2 and 3; Supplemental Tables S2, S3 and S4).

### Comparison of tyrosol responsive genes to farnesol-associated genes

**Fig. 4:**
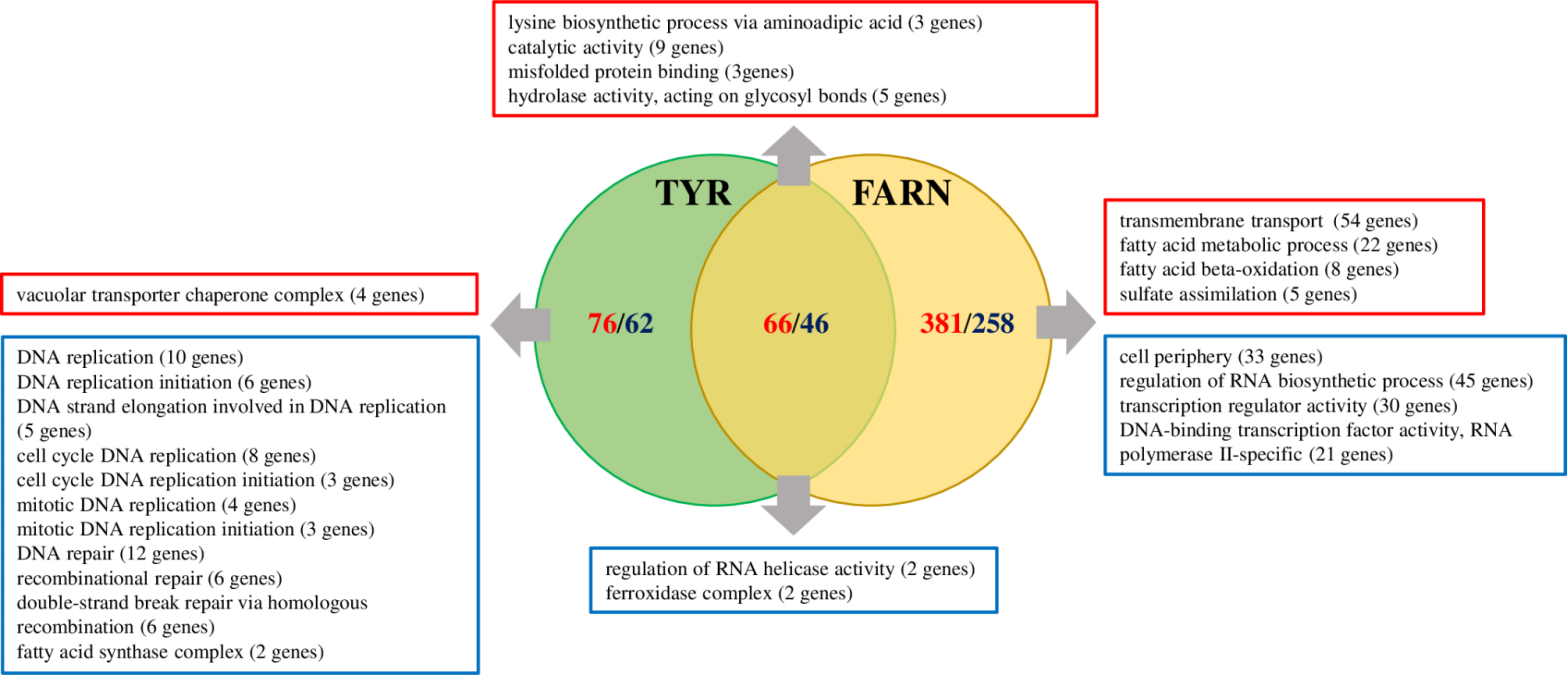
Summary of RNA-Seq data and gene enrichment analyses in case of farnesol and tyrosol exposed *Candida auris* cultures. The effects of tyrosol and farnesol treatment to the transcriptomes are depicted in the Venn diagram, where farnesol-related data derived from the paper published by Jakab et al. (2021). Blue colors shows the down-regulated genes, while red color demonstrates the up-regulated gene groups. Only the differentially expressed genes (corrected *p* value of < 0.05) exhibiting more than 1.5-fold increase or decrease in their transcription are shown. The full list of the gene groups is available in Supplementary Table S2

Farnesol-associated effects were more pronounced on *C. auris* planktonic cells compared to untreated control sessile cells (Jakab et al. 2021). The number of up-regulated genes were 381 and 76 for farnesol and tyrosol, respectively; while 258 and 62 genes were down-regulated for farnesol and tyrosol, respectively (Fig. 4) (Jakab et al. 2021). The overlaps between tyrosol- and farnesol-responsive genes were considerable (66 and 46 overlapping up-regulated and down-regulated genes, respectively) (Fig. 4). It is noteworthy, that several DNA replication-associated genes were down-regulated exclusively under tyrosol treatment. Interestingly, farnesol exposure significantly up-regulated genes related to transmembrane transport, fatty acid metabolic process, fatty acid beta-oxidation and sulfate assimilation (Fig. 4) (Jakab et al. 2021).

## Discussion

In the last two decades, fungal quorum-sensing molecules and related alternative therapeutic solutions have become an intensely researched field (Gupta et al. 2018; Mehmood et al. 2019; Kovács et al. 2020; Costa et al. 2021). However, published results mainly focused on farnesol-associated effects, particularly in the case of *C. albicans*. Available data regarding non-*albicans* and other emerging fungal species (i.e., *C. auris*) remain more scarce. Although *C. auris* has attracted much scientific attention concerning quorum-sensing molecules associated with physiology and antifungal effects, there are still many questions to be answered, especially at the molecular level (Nagy et al. 2020/a,b; Jakab et al. 2021). To date, no data on the physiological effects and the molecular events exerted by tyrosol were available. Nevertheless, metabolic profiling of *C. auris* shows that tyrosol may have a remarkable effect on its physiology, although these effects are still largely unclear (Semreen et al. 2019).

In this study, tyrosol treatment significantly inhibited the growth rate of *C. auris* cells after exposure for 2 h. A similar pattern was detected previously in the case of *C. parapsilosis*, where the observed inhibitory effect was associated with down-regulated ribosome biogenesis genes (Jakab et al. 2019). Interestingly, exposure to 15 mM tyrosol did not significantly change the transcription levels of ribosome biogenesis genes in *C. auris*; however, several genes associated with DNA replication were down-regulated, which may explain the molecular background of the growth inhibition observed. A similar growth inhibition was reported by Cordeiro et al. (2015) at concentrations ranging from 2.5 to 5.0 mM in other non-*albicans* species, such as *C. tropicalis* and *Candida glabrata*. Adampour et al. (2022) showed a significant damage in the cell wall, cell membrane, cytoplasm, nucleus and mitochondria in tyrosol treated planktonic *C. glabrata* cells (Adampour et al. 2022). Regarding non-*Candida* fungal species, tyrosol was able to decrease the ergosterol content of *Coccidioides posadasii* and *Histoplasma capsulatum*; furthermore, it caused an extensive leakage of intracellular molecules (Brilhante et al. 2016). Similar inhibitory effects of tyrosol were reported in case of *Sporothrix* spp. in both filamentous and yeast forms (Brilhante et al. 2020).

The decreased growth rate may be further explained by the increased level of reactive species in the presence of tyrosol. Several previous studies demonstrated that the usage of fungal quorum-sensing molecules at supraphysiological concentrations generates oxidative stress. In addition, there are more differences between farnesol- and tyrosol-related effects on oxidative stress in different *Candida* species (Jakab et al. 2019; Nagy et al. 2020/b). Moreover, *C. auris* has a unique stress resistance profile compared to other *Candida* species, as published previously by Day et al. (2018). In the current study, the increased oxidative stress is associated with elevated activities of catalase, superoxide dismutase and glutathione peroxidase. Nevertheless, the number of up-regulated antioxidative defence-related genes was significantly lower compared to those in *C. parapsilosis* following tyrosol treatment (6 versus 18 genes) (Jakab et al. 2019). Notably, *SOD4* was significantly down-regulated; it is a key gene encoding detoxifying enzymes of *C. albicans* and up-regulated in bloodstream infections (Dantas et al. 2015; Jakab et al. 2019; Allert et al. 2022). This may explain the decreased *Candida* virulence observed in vivo in the case of daily tyrosol treatment (Jakab et al. 2019). In the current study, *CAT1* was up-regulated following exposure to tyrosol in *C. auris*, similar to *C. parapsilosis* (Dantas et al. 2015; Jakab et al. 2019). Based on previous studies, a strong correlation between the expression level of *CAT1* and resistance to hydrogen peroxide was observed in *C. albicans* after farnesol exposure, suggesting that the increase in resistance to oxidative stress in the case of farnesol treatment is correlated with an induction of catalase and that *CAT1* transcription level is a good marker of oxidative stress resistance. Therefore, it is likely that the enhancement of *CAT1* transcription by tyrosol is predictive of the activation of preventive mechanisms against oxidative stress (Deveau et al. 2010). Moreover, *CAP1* was also up-regulated following tyrosol exposure, which is the major regulator transcription factor with regard to oxidative stress elimination (Day et al. 2018; Jakab et al. 2019). The importance of oxidative stress in antifungal effects is further confirmed by the significant decrease in intracellular iron, manganese, copper and zinc contents, which play crucial roles in the protection against oxidative stress. The down-regulated iron uptake genes (*CFL4*, *FRE9*, *FRE10*, *FTR1* and *FTR2*) resulted in a reduced iron content, which may be a part of the general defence strategy to minimise the harmful effect caused by ferrous ions (Dixon et al. 2014). Manganese is a crucial antioxidant element in *Candida* species, whereas the decreased zinc and copper contents prevent the proper function of superoxide dismutase 1, where these elements are structural components (Ballou and Wilson 2016; Gerwien et al. 2018). In addition, the adaptive responses of *Candida* species highly depend on the zinc and copper concentrations, especially upon transitions between commensal, environmental and infective stages (Ballou and Wilson 2016).

Focusing on virulence, generally, a marked down-regulation was observed with regard to genes encoding different virulence factors. Monteiro et al. (2015) reported that tyrosol negatively influence the adhesion. Total metabolic activity profiling revealed that the tyrosol treatment promoted significant reductions (ranging from 22.32 to 51.78%) in the case of *C. albicans* (Monteiro et al. 2015). Surprisingly, in the current study, genes involved in adhesion (*ALS4*, *ALS9* and *IFF4)* were significantly up-regulated. In *C. albicans*, *IFF4* is involved in in vitro and in vivo adherence. Fox et al. (2015), based on transcriptional studies, report that *IFF4* is induced at the later stages of biofilm formation. Notably, a Iff4Δ null mutant exhibited decreased adhesion at an initial stage of biofilm development (Kempf et al. 2009; Kean et al. 2018). Tyrosol has a pivotal role in *C. albicans* morphogenesis and biofilm formation; however, its role in *C. auris* biofilm development remains to be elucidated (Alem et al. 2006). In this study, tyrosol treatment exerted minor changes regarding biofilm-related genes, and based on the obtained results, it mainly promotes later biofilm events. The transcription level of the *RBT1* gene, which promotes biofilm formation in *C. auris*, was increased following tyrosol exposure; however, the ortholog of its regulator of the transcription factor *BCR1* was significantly down-regulated, which was different to the *C. parapsilosis* tyrosol-related response (Ding et al. 2007; Jakab et al. 2019). Another remarkable difference compared to the previously determined *C. parapsilosis* transcription profile was that the *NRG1* (a repressor of hyphal development) gene was down-regulated, stimulating the development of hyphal elements (Ding et al. 2007; Jakab et al. 2019). In addition, it was associated with the increased transcription of *ALS4* (a GPI-anchored protein adhesin), which was up-regulated in filamentous cells of *C. auris*, as published by Yue et al. (2018). The RNA-Seq analysis revealed that the transcription of two major fatty acid biosynthesis-related genes was down-regulated (*FAS1* and *FAS2*); these genes are associated with decreased pathogenesis and virulence (Begum et al. 2022). In our previous study, tyrosol treatment decreased the transcription of genes encoding delta12-fatty acid desaturase (*FAD2*) and omega-3 fatty acid desaturase (*FAD3*), thereby decreasing the general fitness of *C. parapsilosis* (Jakab et al. 2019). Therefore, tyrosol may have a general fatty acid metabolism interfering effect, which can be potentially exploited in novel antifungal strategies targeting this metabolic pathway (DeJarnette et al. 2021).

This is the first study evaluating the global changes in gene transcription in *C. auris* following tyrosol exposure. Analyses of the transcriptional response of this species to tyrosol have revealed several fundamental aspects with regard to quorum-sensing molecule-associated effects. The antifungal effect may be elucidated by the increased oxidative stress and the related reduction in the concentrations of certain intracellular microelements, along with the negative impacts on fatty acid biosynthesis, nucleic acid synthesis and adhesion. Further analysis of these data will not only advance our understanding of the basis of antifungal effects exerted by tyrosol but can also facilitate the development of novel innovative antifungal strategies for the effective treatment of this emerging pathogen.

## Electronic supplementary material

Below is the link to the electronic supplementary material.

Supplementary Material 1

## Data Availability

Regarding the *C. auris* isolate tested, this Whole Genome Shotgun project has been deposited at DDBJ/ENA/GenBank under the accession JANPVY000000000. Transcriptome data have been deposited in NCBI’s Gene Expression Omnibus (GEO; http://www.ncbi.nlm.nih.gov/geo/) and are accessible through GEO Series accession number GSE223953 (https://www.ncbi.nlm.nih.gov/geo/query/acc.cgi?acc=GSE223953).
