# Supplementary material for: Total transcriptome analysis of Candida auris planktonic cells exposed to tyrosol
Source: AMB Express. 2023 Aug 2;13:81. doi: 10.1186/s13568-023-01586-z (PMC10397170; doi:10.1186/s13568-023-01586-z)
Supplement: Supplementary file 1 — Supplementary Material 1 [file 13568_2023_1586_MOESM1_ESM.pdf]

Supplemental Material

Total transcriptome analysis of *Candida auris* planktonic cells exposed to tyrosol

Noémi Balla[1,2], Ágnes Jakab[1,3], Fruzsina Kovács[1,2], Ágota Ragyák[4], Zoltán Tóth[1], Dávid Balázsi[1,2], Lajos Forgács[1,2], Aliz Bozó[1], Farah Al Refai [1], Andrew M Borman[5,6], László Majoros[1], Renátó Kovács[1*]

[1]Department of Medical Microbiology, Faculty of Medicine, University of Debrecen, Debrecen, Hungary

[2]Doctoral School of Pharmaceutical Sciences, University of Debrecen, 4032 Debrecen, Hungary

[3]Department of Molecular Biotechnology and Microbiology, Institute of Biotechnology, Faculty of Science and Technology, University of Debrecen, Debrecen, Hungary.

[4]Department of Inorganic and Analytical Chemistry, Agilent Atomic Spectroscopy Partner Laboratory, University of Debrecen, Debrecen, Hungary

[5]UK National Mycology Reference Laboratory, Public Health England, Science Quarter, Southmead Hospital, Bristol BS10 5NB, UK

[6]Medical Research Council Centre for Medical Mycology (MRCCMM), University of Exeter, Exeter EX4 4QD, UK

*Corresponding author: Renátó Kovács; Department of Medical Microbiology, Faculty of Medicine, University of Debrecen, 4032 Debrecen, Nagyerdei krt. 98., Hungary, Phone: 00-36-52-255-425; e-mail: kovacs.renato@med.unideb.hu (ORCID: 0000-0003-3946-2424)

A

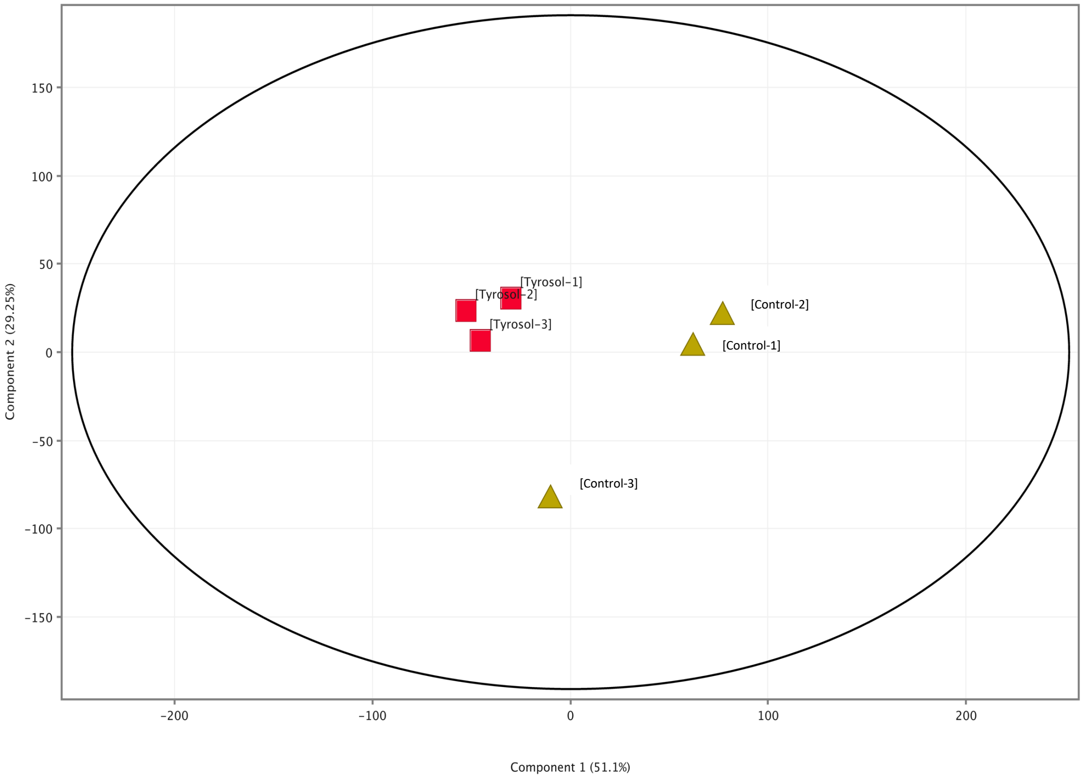

B

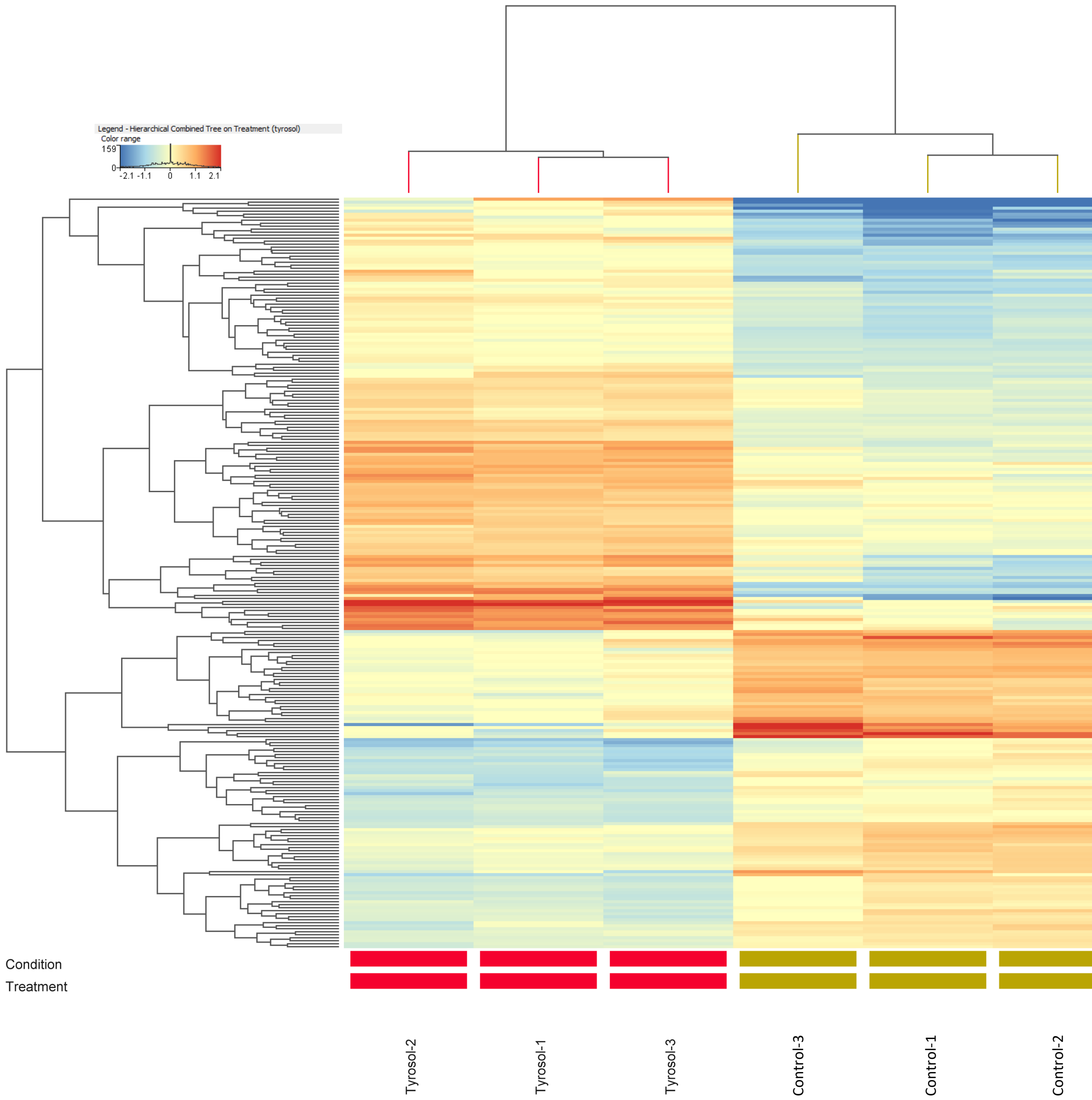

Supplementary Figure 1: Principal component analysis of the transcriptome data (A) and clusters (B).

Symbols represent untreated control (Cont) and 15 mM tyrosol exposure (Tyr) cultures. Analyses were performed with the StrandNGS software using default settings.

Supplementary Tables

Supplementary Table 1: Oligonucleotide primers used for RT-qPCR analysis.

Genes	Forward primer (5'-3')	Reverse primer (5'-3')
ACT1 (B9J08_000486)	CTTGTTCCCAGGTATTGC	CTCGTCGTATTCTTGCTTG
SOD4 (B9J08_001292)	TTACCACGGCTACGAAAAGG	GCACCACCACAGACAAGTTG
CIP1 (B9J08_003024)	GTTGGTGTGGATGAAAGC	AATCGTGCCTGAGTTGAC
CAT1 (B9J08_002298)	GTGCCTACGGTGTCTTTG	TTGGATGGGTCTCTGATG
HSP21 (B9J08_003627)	GCTTGCTGGAAGAAACACC	GACCGCCTTGTCAAAACC
LIP5 (B9J08_004173)	GTTGGTGCTGCTTTCTCC	AATGCTCACGGTTTCCTC
NRG1 (B9J08_005429)	GTGCCTTATCAGAACATTGC	GCCCAGTGTAGTGAACAGAC
HYR1 (B9J08_004098)	GGATTGGCTGCTTCTGTC	CCTTCGTTGTTCACTTTGC
RBT1 (B9J08_001458)	AGGTAACGGTGGCAAAGGTG	AAGGCAGCAGCAAGCAAAG
CDR1 (B9J08_000164)	TTGGAGATGGGTGCTTAC	GCTGAAGGTTGATGGATG
FTR1 (B9J08_002108)	ACGGTTCTGTCCTTTCCTAC	CTTCTTGCCCTTGATATGG
PDC11 (B9J08_002231)	TGTTTGTCGGTGATGGTTC	TGGCTGGATGTCGTTGTAG
ERG1 (B9J08_000261)	GCATTGTACTCGTTATTCGC	ACCCTTCTCGCAGTAGTTG
PFK1 (B9J08_004309)	CGCCTACTCTTCTTTGGAC	ACGGTGGTGTTTCTCTTTC
ADH1 (B9J08_004331)	CTCGCTGCTCCCATCTTG	TCGTCACCACCGTCAATG
FAS1 (B9J08_001296)	GAGCAAGGTATGGGTATGGAC	GGAGAAAGGAAGGTGTAGGAAG
FAS2 (B9J08_002420)	CGTCCACATCCCTCATTG	TCCTTAGCGTCGTTATCGG

Supplementary Table 2. Results of the gene set enrichment analysis

Significant shared GO terms ($p < 0.05$) were determined with Candida Genome Database Gene Ontology Term Finder (http://www.candidagenome.org/cgi-bin/GO/goTermFinder).

Up-regulated and down-regulated genes were defined as differentially expressed genes (p adj. <0.05) where the FoldChange > 1.5, and FoldChange < -1.5, respectively.

Biological processes, molecular function and cellular component categories are provided.

Only terms where the adjusted p value was less than 0.05 were considered as significant enriched terms.

Up-regulated gene set

Biological process

GOID	GO_term	Cluster frequency	Background frequency	Corrected *p* -value	False discovery rate
8652	cellular amino acid biosynthetic process	10 out of 142 genes, 7.0%	89 out of 5417 background genes, 1.6%	0.03356	20.00%
43436	oxoacid metabolic process	20 out of 142 genes, 14.1%	313 out of 5417 background genes, 5.8%	0.05694	8.00%
6797	polyphosphate metabolic process	4 out of 142 genes, 2.8%	12 out of 5417 background genes, 0.2%	0.06601	6.50%

Molecular function

GOID	GO_term	Cluster frequency	Background frequency	Corrected *p* -value	False discovery rate
3824	catalytic activity	9 out of 142 genes, 6.3%	51 out of 5417 background genes, 0.9%	0.00065	0.00%
51787	misfolded protein binding	3 out of 142 genes, 2.1%	6 out of 5417 background genes, 0.1%	0.04061	4.00%

Cellular component

GOID	GO_term	Cluster frequency	Background frequency	Corrected *p* -value	False discovery rate
33254	vacuolar transporter chaperone complex	3 out of 142 genes, 2.1%	4 out of 5417 background genes, 0.1%	0.00484	0.00%

Biological process

GOID	GO_term	Gene(s) annotated to the term
8652	cellular amino acid biosynthetic process	B9J08_000686:B9J08_001277:B9J08_001648:B9J08_001756:B9J08_003103:B9J08_003205:B9J08_003531:B9J08_003722:B9J08_005231:B9J08_005247
43436	oxoacid metabolic process	B9J08_000686:B9J08_000694:B9J08_000871:B9J08_001225:B9J08_001277:B9J08_001464:B9J08_001648:B9J08_001756:B9J08_001883:B9J08_002891:B9J08_003103:B9J08_003205:B9J08_003531:B9J08_003722:B9J08_004292:B9J08_004343:B9J08_004847:B9J08_004930:B9J08_005231:B9J08_005247
6797	polyphosphate metabolic process	B9J08_001225:B9J08_001464:B9J08_004343:B9J08_004847

Molecular function

GOID	GO_term	Gene(s) annotated to the term
3824	catalytic activity	B9J08_000169:B9J08_000266:B9J08_000362:B9J08_000389:B9J08_000512:B9J08_000685:B9J08_000686:B9J08_000694:B9J08_000724:B9J08_000821:B9J08_000834:B9J08_000846:B9J08_000871:B9J08_001012:B9J08_001043:B9J08_001103:B9J08_001116:B9J08_001143:B9J08_001153:B9J08_001225:B9J08_001277:B9J08_001317:B9J08_001339:B9J08_001382:B9J08_001383:B9J08_001405:B9J08_001458:B9J08_001529:B9J08_001532:B9J08_001622:B9J08_001648:B9J08_001659:B9J08_001672:B9J08_001673:B9J08_001702:B9J08_001725:B9J08_001738:B9J08_001756:B9J08_001873:B9J08_001883:B9J08_001898:B9J08_001899:B9J08_001950:B9J08_001976:B9J08_001977:B9J08_001985:B9J08_001991:B9J08_002118:B9J08_002149:B9J08_002298:B9J08_002309:B9J08_002372:B9J08_002383:B9J08_002408:B9J08_002413:B9J08_002457:B9J08_002479:B9J08_002537:B9J08_002538:B9J08_002542:B9J08_002587:B9J08_002643:B9J08_002669:B9J08_002875:B9J08_002878:B9J08_002968:B9J08_003024:B9J08_003056:B9J08_003103:B9J08_003181:B9J08_003205:B9J08_003251:B9J08_003283:B9J08_003370:B9J08_003441:B9J08_003531:B9J08_003595:B9J08_003627:B9J08_003722:B9J08_003723:B9J08_003838:B9J08_004098:B9J08_004176:B9J08_004260:B9J08_004272:B9J08_004292:B9J08_004343:B9J08_004410:B9J08_004476:B9J08_004498:B9J08_004514:B9J08_004515:B9J08_004536:B9J08_004741:B9J08_004828:B9J08_004840:B9J08_004872:B9J08_004930:B9J08_004941:B9J08_004971:B9J08_004974:B9J08_005062:B9J08_005227:B9J08_005231:B9J08_005247:B9J08_005264:B9J08_005308:B9J08_005430
51787	misfolded protein binding	B9J08_000236:B9J08_000476:B9J08_004285

Cellular component

GOID	GO_term	Gene(s) annotated to the term
33254	vacuolar transporter chaperone complex	B9J08_001225:B9J08_004343:B9J08_004847

Down-regulated gene set

Biological process

GOID	GO_term	Cluster frequency	Background frequency	Corrected *p* -value	False discovery rate
51095	regulation of helicase activity	5 out of 108 genes, 4.6%	8 out of 5417 background genes, 0.1%	6.88e-05	0.00%
1902299	pre-replicative complex assembly involved in cell cycle DNA replication	6 out of 108 genes, 5.6%	18 out of 5417 background genes, 0.3%	0.00037	0.00%
36388	pre-replicative complex assembly	6 out of 108 genes, 5.6%	18 out of 5417 background genes, 0.3%	0.00037	0.00%
6267	pre-replicative complex assembly involved in nuclear cell cycle DNA replication	6 out of 108 genes, 5.6%	18 out of 5417 background genes, 0.3%	0.00037	0.00%
33260	nuclear DNA replication	8 out of 108 genes, 7.4%	47 out of 5417 background genes, 0.9%	0.00142	0.00%
44786	cell cycle DNA replication	8 out of 108 genes, 7.4%	47 out of 5417 background genes, 0.9%	0.00142	0.00%
32508	DNA duplex unwinding	5 out of 108 genes, 4.6%	16 out of 5417 background genes, 0.3%	0.00472	0.00%
43462	regulation of ATPase activity	5 out of 108 genes, 4.6%	17 out of 5417 background genes, 0.3%	0.00659	0.00%
32392	DNA geometric change	5 out of 108 genes, 4.6%	18 out of 5417 background genes, 0.3%	0.00898	0.00%
6261	DNA-dependent DNA replication	10 out of 108 genes, 9.3%	100 out of 5417 background genes, 1.8%	0.01133	0.00%
6270	DNA replication initiation	6 out of 108 genes, 5.6%	34 out of 5417 background genes, 0.6%	0.02095	0.00%
6260	DNA replication	10 out of 108 genes, 9.3%	108 out of 5417 background genes, 2.0%	0.02219	0.17%
51097	negative regulation of helicase activity	3 out of 108 genes, 2.8%	5 out of 5417 background genes, 0.1%	0.03362	0.31%
727	double-strand break repair via break-induced replication	5 out of 108 genes, 4.6%	24 out of 5417 background genes, 0.4%	0.04050	0.29%
6271	DNA strand elongation involved in DNA replication	5 out of 108 genes, 4.6%	25 out of 5417 background genes, 0.5%	0.04982	0.27%

Molecular function

GOID	GO_term	Cluster frequency	Background frequency	Corrected *p* -value	False discovery rate
17116	single-stranded DNA helicase activity	5 out of 108 genes, 4.6%	9 out of 5417 background genes, 0.2%	3.12e-05	0.00%
3688	DNA replication origin binding	7 out of 108 genes, 6.5%	29 out of 5417 background genes, 0.5%	0.00010	0.00%
1990518	single-stranded 3'-5' DNA helicase activity	3 out of 108 genes, 2.8%	3 out of 5417 background genes, 0.1%	0.00070	0.00%
33679	3'-5' DNA/RNA helicase activity	3 out of 108 genes, 2.8%	3 out of 5417 background genes, 0.1%	0.00070	0.00%
9378	four-way junction helicase activity	3 out of 108 genes, 2.8%	5 out of 5417 background genes, 0.1%	0.00688	0.40%
3727	single-stranded RNA binding	5 out of 108 genes, 4.6%	30 out of 5417 background genes, 0.6%	0.02529	1.00%
3678	DNA helicase activity	5 out of 108 genes, 4.6%	32 out of 5417 background genes, 0.6%	0.03463	1.14%
33677	DNA/RNA helicase activity	3 out of 108 genes, 2.8%	8 out of 5417 background genes, 0.1%	0.03692	2.00%

Cellular component

GOID	GO_term	Cluster frequency	Background frequency	Corrected *p* -value	False discovery rate
42555	MCM complex	5 out of 108 genes, 4.6%	5 out of 5417 background genes, 0.1%	2.93e-07	0.00%
36387	pre-replicative complex	6 out of 108 genes, 5.6%	16 out of 5417 background genes, 0.3%	3.79e-05	0.00%
5656	nuclear pre-replicative complex	6 out of 108 genes, 5.6%	16 out of 5417 background genes, 0.3%	3.79e-05	0.00%
71162	CMG complex	5 out of 108 genes, 4.6%	10 out of 5417 background genes, 0.2%	6.81e-05	0.00%
31261	DNA replication preinitiation complex	6 out of 108 genes, 5.6%	21 out of 5417 background genes, 0.4%	0.00023	0.00%
31298	replication fork protection complex	6 out of 108 genes, 5.6%	21 out of 5417 background genes, 0.4%	0.00023	0.00%
97373	MCM core complex	3 out of 108 genes, 2.8%	3 out of 5417 background genes, 0.1%	0.00078	0.00%
5657	replication fork	8 out of 108 genes, 7.4%	53 out of 5417 background genes, 1.0%	0.00082	0.00%
43596	nuclear replication fork	7 out of 108 genes, 6.5%	40 out of 5417 background genes, 0.7%	0.00113	0.00%
32993	protein-DNA complex	7 out of 108 genes, 6.5%	54 out of 5417 background genes, 1.0%	0.00860	0.00%
5835	fatty acid synthase complex	2 out of 108 genes, 1.9%	2 out of 5417 background genes, 0.0%	0.04017	0.91%

Biological process

GOID	GO_term	Gene(s) annotated to the term
51095	regulation of helicase activity	B9J08_000087:B9J08_003467:B9J08_005188:B9J08_005385:B9J08_005476
1902299	pre-replicative complex assembly involved in cell cycle DNA replication	B9J08_000087:B9J08_001105:B9J08_002424:B9J08_003467:B9J08_003937:B9J08_005476
36388	pre-replicative complex assembly	B9J08_000087:B9J08_001105:B9J08_002424:B9J08_003467:B9J08_003937:B9J08_005476
6267	pre-replicative complex assembly involved in nuclear cell cycle DNA replication	B9J08_000087:B9J08_001105:B9J08_002424:B9J08_003467:B9J08_003937:B9J08_005476
33260	nuclear DNA replication	B9J08_000087:B9J08_001105:B9J08_002424:B9J08_003467:B9J08_003937:B9J08_005422:B9J08_005476:B9J08_005515
44786	cell cycle DNA replication	B9J08_000087:B9J08_001105:B9J08_002424:B9J08_003467:B9J08_003937:B9J08_005422:B9J08_005476:B9J08_005515
32508	DNA duplex unwinding	B9J08_000087:B9J08_001105:B9J08_002424:B9J08_003467:B9J08_005476
43462	regulation of ATPase activity	B9J08_000087:B9J08_003467:B9J08_005188:B9J08_005385:B9J08_005476
32392	DNA geometric change	B9J08_000087:B9J08_001105:B9J08_002424:B9J08_003467:B9J08_005476
6261	DNA-dependent DNA replication	B9J08_000087:B9J08_001105:MRC1:B9J08_002424:B9J08_003338:B9J08_003467:B9J08_003937:B9J08_005422:B9J08_005476:B9J08_005515
6270	DNA replication initiation	B9J08_000087:B9J08_001105:B9J08_002424:B9J08_003467:B9J08_005476:B9J08_005515
6260	DNA replication	B9J08_000087:B9J08_001105:MRC1:B9J08_002424:B9J08_003338:B9J08_003467:B9J08_003937:B9J08_005422:B9J08_005476:B9J08_005515
51097	negative regulation of helicase activity	B9J08_000087:B9J08_003467:B9J08_005476
727	double-strand break repair via break-induced replication	B9J08_000087:B9J08_001105:B9J08_002424:B9J08_003467:B9J08_005476
6271	DNA strand elongation involved in DNA replication	B9J08_001105:B9J08_002424:B9J08_003338:B9J08_003467:B9J08_005476

Molecular function

GOID	GO_term	Gene(s) annotated to the term
17116	single-stranded DNA helicase activity	B9J08_000087:B9J08_001105:B9J08_002424:B9J08_003467:B9J08_005476
3688	DNA replication origin binding	B9J08_000087:B9J08_001105:B9J08_002424:B9J08_003467:B9J08_003937:B9J08_005476:B9J08_005515
1990518	single-stranded 3'-5' DNA helicase activity	B9J08_001105:B9J08_002424:B9J08_003467

33679	3'-5' DNA/RNA helicase activity	B9J08_001105:B9J08_002424:B9J08_003467
9378	four-way junction helicase activity	B9J08_001105:B9J08_002424:B9J08_003467
3727	single-stranded RNA binding	B9J08_000774:B9J08_001105:B9J08_002424:B9J08_003467:B9J08_005385
3678	DNA helicase activity	B9J08_000087:B9J08_001105:B9J08_002424:B9J08_003467:B9J08_005476
33677	DNA/RNA helicase activity	B9J08_001105:B9J08_002424:B9J08_003467

Cellular component

GOID	GO_term	Gene(s) annotated to the term
42555	MCM complex	B9J08_000087:B9J08_001105:B9J08_002424:B9J08_003467:B9J08_005476
36387	pre-replicative complex	B9J08_000087:B9J08_001105:B9J08_002424:B9J08_003467:B9J08_003937:B9J08_005476
5656	nuclear pre-replicative complex	B9J08_000087:B9J08_001105:B9J08_002424:B9J08_003467:B9J08_003937:B9J08_005476
71162	CMG complex	B9J08_000087:B9J08_001105:B9J08_002424:B9J08_003467:B9J08_005476
31261	DNA replication preinitiation complex	B9J08_000087:B9J08_001105:B9J08_002424:B9J08_003467:B9J08_003937:B9J08_005476
31298	replication fork protection complex	B9J08_000087:B9J08_001105:MRC1:B9J08_002424:B9J08_003467:B9J08_005476
97373	MCM core complex	B9J08_001105:B9J08_002424:B9J08_003467
5657	replication fork	B9J08_000087:B9J08_001105:MRC1:B9J08_002424:B9J08_003338:B9J08_003467:B9J08_005476:B9J08_005515
43596	nuclear replication fork	B9J08_000087:B9J08_001105:MRC1:B9J08_002424:B9J08_003467:B9J08_005476:B9J08_005515
32993	protein-DNA complex	B9J08_000087:B9J08_001105:B9J08_002424:B9J08_003467:B9J08_003937:B9J08_005476:B9J08_005515
5835	fatty acid synthase complex	B9J08_001296:B9J08_002420

Supplementary Table 2. Results of the gene set enrichment analysis

Up-regulated and down-regulated genes under Tyrosol or Farnesol treatment conditions.

Up-regulated and down-regulated genes were defined as differentially expressed genes (*p* adj. <0.05) where the FoldChange > 1.5, and FoldChange < -1.5, respectively.

Biological processes, molecular function and cellular component categories are provided.

Only terms where the adjusted *p* value was less than 0.05 were considered as significant enriched terms.

Up-regulated gene set

The overlaps between Tyrosol and Farnesol responsive genes

Biological process

GOID	GO_term	Cluster frequency	Background frequency	Corrected *p* -value	False discovery rate
19878	lysine biosynthetic process via aminoadipic acid	3 out of 66 genes, 4.5%	7 out of 5417 background genes, 0.1%	0.00911	0.00%
6553	lysine metabolic process	3 out of 66 genes, 4.5%	9 out of 5417 background genes, 0.2%	0.02148	3.00%
9085	lysine biosynthetic process	3 out of 66 genes, 4.5%	9 out of 5417 background genes, 0.2%	0.02148	2.00%

Molecular function

GOID	GO_term	Cluster frequency	Background frequency	Corrected *p* -value	False discovery rate
3824	catalytic activity	9 out of 66 genes, 13.6%	56 out of 5417 background genes, 1.0%	1.25E-06	0.00%
51787	misfolded protein binding	3 out of 66 genes, 4.5%	6 out of 5417 background genes, 0.1%	0.00259	0.00%
4553	hydrolase activity, hydrolyzing O-glycosyl compounds	5 out of 66 genes, 7.6%	36 out of 5417 background genes, 0.7%	0.00499	0.00%
16798	hydrolase activity, acting on glycosyl bonds	5 out of 66 genes, 7.6%	46 out of 5417 background genes, 0.8%	0.01652	3.00%

Significantly enriched gene group in Tyrosol treated cultures

Cellular component

GOID	GO_term	Cluster frequency	Background frequency	Corrected *p* -value	False discovery rate
33254	vacuolar transporter chaperone complex	3 out of 76 genes, 3.9%	4 out of 5417 background genes, 0.1%	0.0004	0.00%

Significantly enriched gene groups in Farnesol treated cultures

Biological process

GOID	GO_term	Cluster frequency	Background frequency	Corrected *p* -value	False discovery rate
9062	fatty acid catabolic process	13 out of 381 genes, 3.4%	17 out of 5417 background genes, 0.3%	9.22E-10	0.00%
6631	fatty acid metabolic process	22 out of 381 genes, 5.8%	58 out of 5417 background genes, 1.1%	7.60E-09	0.00%
19395	fatty acid oxidation	10 out of 381 genes, 2.6%	11 out of 5417 background genes, 0.2%	1.61E-08	0.00%
34440	lipid oxidation	10 out of 381 genes, 2.6%	11 out of 5417 background genes, 0.2%	1.61E-08	0.00%
16042	lipid catabolic process	18 out of 381 genes, 4.7%	43 out of 5417 background genes, 0.8%	8.43E-08	0.00%
44242	cellular lipid catabolic process	16 out of 381 genes, 4.2%	34 out of 5417 background genes, 0.6%	1.06E-07	0.00%
6635	fatty acid beta-oxidation	8 out of 381 genes, 2.1%	9 out of 5417 background genes, 0.2%	2.79E-06	0.00%
72329	monocarboxylic acid catabolic process	14 out of 381 genes, 3.7%	33 out of 5417 background genes, 0.6%	8.09E-06	0.00%
55085	transmembrane transport	54 out of 381 genes, 14.2%	358 out of 5417 background genes, 6.6%	1.92E-05	0.00%
32787	monocarboxylic acid metabolic process	27 out of 381 genes, 7.1%	123 out of 5417 background genes, 2.3%	3.77E-05	0.00%
30258	lipid modification	10 out of 381 genes, 2.6%	19 out of 5417 background genes, 0.4%	8.13E-05	0.00%
19752	carboxylic acid metabolic process	46 out of 381 genes, 12.1%	314 out of 5417 background genes, 5.8%	0.00053	0.00%
43436	oxoacid metabolic process	46 out of 381 genes, 12.1%	326 out of 5417 background genes, 6.0%	0.00157	0.00%
6082	organic acid metabolic process	46 out of 381 genes, 12.1%	327 out of 5417 background genes, 6.0%	0.00171	0.00%
16054	organic acid catabolic process	15 out of 381 genes, 3.9%	64 out of 5417 background genes, 1.2%	0.01505	0.12%
46395	carboxylic acid catabolic process	15 out of 381 genes, 3.9%	64 out of 5417 background genes, 1.2%	0.01505	0.12%
103	sulfate assimilation	5 out of 381 genes, 1.3%	8 out of 5417 background genes, 0.1%	0.04657	0.78%

Molecular function

GOID	GO_term	Cluster frequency	Background frequency	Corrected *p* -value	False discovery rate
16491	oxidoreductase activity	53 out of 381 genes, 13.9%	346 out of 5417 background genes, 6.4%	5.33E-06	0.00%
22857	transmembrane transporter activity	48 out of 381 genes, 12.6%	359 out of 5417 background genes, 6.6%	0.00151	0.00%
5215	transporter activity	50 out of 381 genes, 13.1%	383 out of 5417 background genes, 7.1%	0.00189	0.00%
8514	organic anion transmembrane transporter activity	13 out of 381 genes, 3.4%	50 out of 5417 background genes, 0.9%	0.00556	0.50%
16712	oxidoreductase activity, acting on paired donors, with incorporation or reduction of molecular oxygen, reduced flavin or flavoprotein as one donor, and incorporation of one atom of oxygen	5 out of 381 genes, 1.3%	8 out of 5417 background genes, 0.1%	0.01612	0.40%

46943	carboxylic acid transmembrane transporter activity	10 out of 381 genes, 2.6%	38 out of 5417 background genes, 0.7%	0.0428	0.86%
5342	organic acid transmembrane transporter activity	10 out of 381 genes, 2.6%	38 out of 5417 background genes, 0.7%	0.0428	0.75%

Cellular component

GOID	GO_term	Cluster frequency	Background frequency	Corrected *p* -value	False discovery rate
42579	microbody	20 out of 381 genes, 5.2%	62 out of 5417 background genes, 1.1%	3.68E-07	0.00%
5777	peroxisome	20 out of 381 genes, 5.2%	62 out of 5417 background genes, 1.1%	3.68E-07	0.00%
31907	microbody lumen	7 out of 381 genes, 1.8%	9 out of 5417 background genes, 0.2%	2.98E-05	0.00%
5782	peroxisomal matrix	7 out of 381 genes, 1.8%	9 out of 5417 background genes, 0.2%	2.98E-05	0.00%
16021	integral component of membrane	38 out of 381 genes, 10.0%	292 out of 5417 background genes, 5.4%	0.01478	0.67%

The overlaps between Tyrosol and Farnesol responsive genes

Biological process

GOID	GO_term	Gene(s) annotated to the term
19878	lysine biosynthetic process via aminoadipic acid	B9J08_001756:B9J08_003205:B9J08_003722
6553	lysine metabolic process	B9J08_001756:B9J08_003205:B9J08_003722
9085	lysine biosynthetic process	B9J08_001756:B9J08_003205:B9J08_003722

Molecular function

GOID	GO_term	Gene(s) annotated to the term
3824	catalytic activity	B9J08_000266:B9J08_000389:B9J08_000476:B9J08_000512:B9J08_000686:B9J08_000724:B9J08_000834:B9J08_000846:B9J08_000871:B9J08_001043:B9J08_001143:B9J08_001277:B9J08_001339:B9J08_001382:B9J08_001383:B9J08_001405:B9J08_001529:B9J08_001622:B9J08_001673:B9J08_001702:B9J08_001756:B9J08_001950:B9J08_001977:B9J08_001985:B9J08_002298:B9J08_002408:B9J08_002413:B9J08_002457:B9J08_002479:B9J08_002587:B9J08_002643:B9J08_002878:B9J08_003181:B9J08_003205:B9J08_003251:B9J08_003595:B9J08_003627:B9J08_003722:B9J08_004260:B9J08_004285:B9J08_004410:B9J08_004476:B9J08_004514:B9J08_004515:B9J08_004741:B9J08_004828:B9J08_004941:B9J08_004971:B9J08_005062:B9J08_005308:B9J08_005430
51787	misfolded protein binding	B9J08_000236:B9J08_000476:B9J08_004285
4553	hydrolase activity, hydrolyzing O-glycosyl compounds	B9J08_001383:B9J08_001529:B9J08_001985:B9J08_003251:B9J08_004410

16798	hydrolase activity, acting on glycosyl bonds	B9J08_001383:B9J08_001529:B9J08_001985:B9J08_003251:B9J08_004410

Significantly enriched gene group in Tyrosol treated cultures

Cellular component

GOID	GO_term	Gene(s) annotated to the term
33254	vacuolar transporter chaperone complex	B9J08_001225:B9J08_004343:B9J08_004847

Significantly enriched gene groups in Farnesol treated cultures

Biological process

GOID	GO_term	Gene(s) annotated to the term
9062	fatty acid catabolic process	B9J08_000010:B9J08_000147:B9J08_001484:B9J08_001740:B9J08_001956:B9J08_002847:B9J08_003185:B9J08_003374:B9J08_003466:B9J08_003628:B9J08_003678:B9J08_004365:B9J08_004905
6631	fatty acid metabolic process	B9J08_000010:B9J08_000147:B9J08_000397:B9J08_001296:B9J08_001484:B9J08_001740:B9J08_001861:B9J08_001889:B9J08_001956:B9J08_002157:B9J08_002488:B9J08_002781:B9J08_002847:B9J08_003185:B9J08_003285:B9J08_003374:B9J08_003462:B9J08_003466:B9J08_003628:B9J08_003678:B9J08_004365:B9J08_004905
19395	fatty acid oxidation	B9J08_000147:B9J08_000397:B9J08_001484:B9J08_001740:B9J08_002847:B9J08_003185:B9J08_003466:B9J08_003628:B9J08_003678:B9J08_004905
34440	lipid oxidation	B9J08_000147:B9J08_000397:B9J08_001484:B9J08_001740:B9J08_002847:B9J08_003185:B9J08_003466:B9J08_003628:B9J08_003678:B9J08_004905
16042	lipid catabolic process	B9J08_000010:B9J08_000147:B9J08_001484:B9J08_001740:B9J08_001956:B9J08_002814:B9J08_002847:B9J08_003185:B9J08_003374:B9J08_003466:B9J08_003628:B9J08_003678:B9J08_004096:B9J08_004111:B9J08_004156:B9J08_004365:B9J08_004905:B9J08_005309
44242	cellular lipid catabolic process	185:B9J08_003374:B9J08_003466:B9J08_003628:B9J08_003678:B9J08_004096:B9J08_004365:B9J08_004905:B9J08_005309
6635	fatty acid beta-oxidation	B9J08_001484:B9J08_001740:B9J08_002847:B9J08_003185:B9J08_003466:B9J08_003628:B9J08_003678:B9J08_004905
72329	monocarboxylic acid catabolic process	B9J08_000010:B9J08_000147:B9J08_001484:B9J08_001740:B9J08_001889:B9J08_001956:B9J08_002847:B9J08_003185:B9J08_003374:B9J08_003466:B9J08_003628:B9J08_003678:B9J08_004365:B9J08_004905

55085	transmembrane transport	B9J08_000033:CDR1:B9J08_000175:B9J08_000321:B9J08_000473:CDR4:B9J08_000483:B9J08_000503:B9J08_000896:B9J08_000982:B9J08_000995:B9J08_001362:B9J08_001523:B9J08_001530:B9J08_001895:B9J08_001952:B9J08_001984:B9J08_002006:B9J08_002087:B9J08_002202:B9J08_002227:B9J08_002239:B9J08_002242:B9J08_002251:B9J08_002297:B9J08_002488:B9J08_002646:B9J08_002659:B9J08_002821:B9J08_003462:B9J08_003465:B9J08_003527:B9J08_003690:B9J08_003734:B9J08_003832:B9J08_003908:MDR1:B9J08_004066:B9J08_004365:B9J08_004429:B9J08_004448:B9J08_004533:B9J08_004537:B9J08_004562:B9J08_004592:B9J08_004602:B9J08_004690:B9J08_004825:B9J08_004878:B9J08_005345:B9J08_005491:B9J08_005564:B9J08_005570:B9J08_005571
32787	monocarboxylic acid metabolic process	B9J08_000010:B9J08_000147:B9J08_000229:B9J08_000397:B9J08_000989:B9J08_001296:B9J08_001484:B9J08_001641:B9J08_001740:B9J08_001861:B9J08_001884:B9J08_001889:B9J08_001956:B9J08_002157:B9J08_002488:B9J08_002781:B9J08_002847:B9J08_003185:B9J08_003285:B9J08_003374:B9J08_003462:B9J08_003466:B9J08_003628:B9J08_003678:B9J08_003998:B9J08_004365:B9J08_004905
30258	lipid modification	B9J08_000147:B9J08_000397:B9J08_001484:B9J08_001740:B9J08_002847:B9J08_003185:B9J08_003466:B9J08_003628:B9J08_003678:B9J08_004905
19752	carboxylic acid metabolic process	B9J08_000010:B9J08_000013:B9J08_000147:B9J08_000229:B9J08_000397:B9J08_000613:B9J08_000755:B9J08_000989:B9J08_001224:B9J08_001296:B9J08_001484:B9J08_001641:B9J08_001645:B9J08_001740:B9J08_001861:B9J08_001884:B9J08_001889:B9J08_001956:B9J08_001967:B9J08_002157:B9J08_002351:B9J08_002375:B9J08_002469:B9J08_002488:B9J08_002781:B9J08_002847:B9J08_002995:B9J08_003167:B9J08_003180:B9J08_003185:B9J08_003285:B9J08_003374:B9J08_003440:B9J08_003462:B9J08_003466:B9J08_003514:B9J08_003628:B9J08_003678:B9J08_003998:B9J08_004365:B9J08_004781:B9J08_004798:B9J08_004905:B9J08_005199:B9J08_005307:B9J08_005516
43436	oxoacid metabolic process	B9J08_000010:B9J08_000013:B9J08_000147:B9J08_000229:B9J08_000397:B9J08_000613:B9J08_000755:B9J08_000989:B9J08_001224:B9J08_001296:B9J08_001484:B9J08_001641:B9J08_001645:B9J08_001740:B9J08_001861:B9J08_001884:B9J08_001889:B9J08_001956:B9J08_001967:B9J08_002157:B9J08_002351:B9J08_002375:B9J08_002469:B9J08_002488:B9J08_002781:B9J08_002847:B9J08_002995:B9J08_003167:B9J08_003180:B9J08_003185:B9J08_003285:B9J08_003374:B9J08_003440:B9J08_003462:B9J08_003466:B9J08_003514:B9J08_003628:B9J08_003678:B9J08_003998:B9J08_004365:B9J08_004781:B9J08_004798:B9J08_004905:B9J08_005199:B9J08_005307:B9J08_005516
6082	organic acid metabolic process	B9J08_000010:B9J08_000013:B9J08_000147:B9J08_000229:B9J08_000397:B9J08_000613:B9J08_000755:B9J08_000989:B9J08_001224:B9J08_001296:B9J08_001484:B9J08_001641:B9J08_001645:B9J08_001740:B9J08_001861:B9J08_001884:B9J08_001889:B9J08_001956:B9J08_001967:B9J08_002157:B9J08_002351:B9J08_002375:B9J08_002469:B9J08_002488:B9J08_002781:B9J08_002847:B9J08_002995:B9J08_003167:B9J08_003180:B9J08_003185:B9J08_003285:B9J08_003374:B9J08_003440:B9J08_003462:B9J08_003466:B9J08_003514:B9J08_003628:B9J08_003678:B9J08_003998:B9J08_004365:B9J08_004781:B9J08_004798:B9J08_004905:B9J08_005199:B9J08_005307:B9J08_005516
16054	organic acid catabolic process	B9J08_000010:B9J08_000147:B9J08_001484:B9J08_001740:B9J08_001889:B9J08_001956:B9J08_001967:B9J08_002847:B9J08_003185:B9J08_003374:B9J08_003466:B9J08_003628:B9J08_003678:B9J08_004365:B9J08_004905
46395	carboxylic acid catabolic process	B9J08_000010:B9J08_000147:B9J08_001484:B9J08_001740:B9J08_001889:B9J08_001956:B9J08_001967:B9J08_002847:B9J08_003185:B9J08_003374:B9J08_003466:B9J08_003628:B9J08_003678:B9J08_004365:B9J08_004905
103	sulfate assimilation	B9J08_001224:B9J08_002351:B9J08_002995:B9J08_003552:B9J08_005307

Molecular function

GOID	GO_term	Gene(s) annotated to the term
16491	oxidoreductase activity	B9J08_000008:B9J08_000147:B9J08_000229:B9J08_000487:B9J08_000568:B9J08_000613:B9J08_000625:B9J08_000755:B9J08_000989:B9J08_001224:B9J08_001296:B9J08_001515:B9J08_001533:B9J08_001550:B9J08_001570:B9J08_001588:B9J08_001660:B9J08_001688:B9J08_001733:B9J08_001740:B9J08_001956:B9J08_001996:B9J08_002011:B9J08_002143:B9J08_002157:B9J08_002254:B9J08_002366:B9J08_002375:B9J08_002419:B9J08_002427:B9J08_002531:B9J08_002668:B9J08_002830:B9J08_002847:B9J08_003167:B9J08_003180:B9J08_003266:B9J08_003267:B9J08_003437:B9J08_003440:B9J08_003442:B9J08_003466:B9J08_003552:B9J08_003628:B9J08_003681:B9J08_003684:B9J08_004114:ERG5:B9J08_004600:B9J08_004656:B9J08_005307:B9J08_005516:B9J08_005548
22857	transmembrane transporter activity	B9J08_000032:B9J08_000033:CDR1:B9J08_000175:B9J08_000321:B9J08_000473:CDR4:B9J08_000503:B9J08_000896:B9J08_000982:B9J08_000995:B9J08_001362:B9J08_001523:B9J08_001530:B9J08_001889:B9J08_001952:B9J08_001984:B9J08_002006:B9J08_002202:B9J08_002227:B9J08_002239:B9J08_002251:B9J08_002297:B9J08_002646:B9J08_002659:B9J08_003465:B9J08_003527:B9J08_003678:B9J08_003832:B9J08_003908:MDR1:B9J08_004066:B9J08_004365:B9J08_004429:B9J08_004448:B9J08_004533:B9J08_004537:B9J08_004562:B9J08_004592:B9J08_004602:B9J08_004690:B9J08_004825:B9J08_004878:B9J08_005345:B9J08_005491:B9J08_005564:B9J08_005570:B9J08_0055
5215	transporter activity	B9J08_000032:B9J08_000033:CDR1:B9J08_000175:B9J08_000321:B9J08_000473:CDR4:B9J08_000503:B9J08_000896:B9J08_000982:B9J08_000995:B9J08_001362:B9J08_001523:B9J08_001530:B9J08_001861:B9J08_001889:B9J08_001952:B9J08_001984:B9J08_002006:B9J08_002202:B9J08_002227:B9J08_002239:B9J08_002251:B9J08_002297:B9J08_002442:B9J08_002646:B9J08_002659:B9J08_003465:B9J08_003527:B9J08_003678:B9J08_003832:B9J08_003908:MDR1:B9J08_004066:B9J08_004365:B9J08_004429:B9J08_004448:B9J08_004533:B9J08_004537:B9J08_004562:B9J08_004592:B9J08_004602:B9J08_004690:B9J08_004825:B9J08_004878:B9J08_005345:B9J08_005491:B9J08_005564:B9J08_005570:B9J08_005571
8514	organic anion transmembrane transporter activity	B9J08_000473:B9J08_000503:B9J08_001362:B9J08_001530:B9J08_002006:B9J08_004066:B9J08_004365:B9J08_004429:B9J08_004448:B9J08_004592:B9J08_005345:B9J08_005491:B9J08_005564
16712	oxidoreductase activity, acting on paired donors, with incorporation or reduction of molecular oxygen, reduced flavin or flavoprotein as one donor, and incorporation of one atom of oxygen	B9J08_001515:B9J08_001588:B9J08_002668:ERG5:B9J08_005548
46943	carboxylic acid transmembrane transporter activity	B9J08_001362:B9J08_001530:B9J08_002006:B9J08_004365:B9J08_004429:B9J08_004448:B9J08_004592:B9J08_005345:B9J08_005491:B9J08_005564
5342	organic acid transmembrane transporter activity	B9J08_001362:B9J08_001530:B9J08_002006:B9J08_004365:B9J08_004429:B9J08_004448:B9J08_004592:B9J08_005345:B9J08_005491:B9J08_005564

Cellular component

GOID	GO_term	Gene(s) annotated to the term

42579	microbody	B9J08_000010:B9J08_000032:B9J08_000397:B9J08_001484:B9J08_001645:B9J08_001740:B9J08_001861:B9J08_001956:B9J08_002488:B9J08_002781:B9J08_002847:B9J08_003180:B9J08_003185:B9J08_003374:B9J08_003442:B9J08_003462:B9J08_003466:B9J08_003628:B9J08_004096:B9J08_004905
5777	peroxisome	B9J08_000010:B9J08_000032:B9J08_000397:B9J08_001484:B9J08_001645:B9J08_001740:B9J08_001861:B9J08_001956:B9J08_002488:B9J08_002781:B9J08_002847:B9J08_003180:B9J08_003185:B9J08_003374:B9J08_003442:B9J08_003462:B9J08_003466:B9J08_003628:B9J08_004096:B9J08_004905
31907	microbody lumen	B9J08_001484:B9J08_001740:B9J08_001956:B9J08_003374:B9J08_003442:B9J08_003466:B9J08_004096
5782	peroxisomal matrix	B9J08_001484:B9J08_001740:B9J08_001956:B9J08_003374:B9J08_003442:B9J08_003466:B9J08_004096
16021	integral component of membrane	B9J08_000033:CDR1:B9J08_000175:B9J08_000321:CDR4:B9J08_000503:B9J08_000625:B9J08_000896:B9J08_000995:B9J08_001523:B9J08_001682:B9J08_001984:B9J08_002087:B9J08_002239:B9J08_002251:B9J08_002297:B9J08_002646:B9J08_002659:B9J08_002817:B9J08_002835:B9J08_003336:B9J08_003465:B9J08_003527:B9J08_003668:B9J08_003832:B9J08_003908:B9J08_003928:MDR1:B9J08_004437:B9J08_004448:B9J08_004533:B9J08_004690:B9J08_005283:B9J08_005309:B9J08_005345:B9J08_005564:B9J08_005570:B9J08_005571

Down-regulated gene set

The overlaps between Tyrosol and Farnesol responsive genes

Biological process

GOID	GO_term	Cluster frequency	Background frequency	Corrected *p* -value	False discovery rate
2E+06	regulation of RNA helicase activity	2 out of 46 genes, 4.3%	3 out of 5417 background genes, 0.1%	0.04378	58.00%

Molecular function

GOID	GO_term	Cluster frequency	Background frequency	Corrected *p* -value	False discovery rate
43024	ribosomal small subunit binding	2 out of 46 genes, 4.3%	4 out of 5417 background genes, 0.1%	0.02554	18.00%

Cellular component

GOID	GO_term	Cluster frequency	Background frequency	Corrected *p* -value	False discovery rate
2E+06	ferroxidase complex	2 out of 46 genes, 4.3%	5 out of 5417 background genes, 0.1%	0.0354	32.00%
33573	high-affinity iron permease complex	2 out of 46 genes, 4.3%	5 out of 5417 background genes, 0.1%	0.0354	16.00%

Significantly enriched gene groups in Tyrosol treated cultures

Biological process

GOID	GO_term	Cluster frequency	Background frequency	Corrected *p* -value	False discovery rate
2E+06	pre-replicative complex assembly involved in cell cycle DNA replication	6 out of 62 genes, 9.7%	18 out of 5417 background genes, 0.3%	6.44E-06	0.00%
36388	pre-replicative complex assembly	6 out of 62 genes, 9.7%	18 out of 5417 background genes, 0.3%	6.44E-06	0.00%
6267	pre-replicative complex assembly involved in nuclear cell cycle DNA replication	6 out of 62 genes, 9.7%	18 out of 5417 background genes, 0.3%	6.44E-06	0.00%
33260	nuclear DNA replication	8 out of 62 genes, 12.9%	46 out of 5417 background genes, 0.8%	7.55E-06	0.00%
44786	cell cycle DNA replication	8 out of 62 genes, 12.9%	46 out of 5417 background genes, 0.8%	7.55E-06	0.00%
6260	DNA replication	10 out of 62 genes, 16.1%	107 out of 5417 background genes, 2.0%	5.94E-05	0.00%
32508	DNA duplex unwinding	5 out of 62 genes, 8.1%	16 out of 5417 background genes, 0.3%	0.00014	0.00%
6261	DNA-templated DNA replication	9 out of 62 genes, 14.5%	96 out of 5417 background genes, 1.8%	0.00024	0.00%
32392	DNA geometric change	5 out of 62 genes, 8.1%	18 out of 5417 background genes, 0.3%	0.00028	0.00%
6270	DNA replication initiation	6 out of 62 genes, 9.7%	34 out of 5417 background genes, 0.6%	0.0004	0.00%
6259	DNA metabolic process	15 out of 62 genes, 24.2%	320 out of 5417 background genes, 5.9%	0.00043	0.00%

6281	DNA repair	12 out of 62 genes, 19.4%	219 out of 5417 background genes, 4.0%	0.00111	0.00%
727	double-strand break repair via break-induced replication	5 out of 62 genes, 8.1%	24 out of 5417 background genes, 0.4%	0.00131	0.00%
6271	DNA strand elongation involved in DNA replication	5 out of 62 genes, 8.1%	25 out of 5417 background genes, 0.5%	0.00163	0.00%
71103	DNA conformation change	5 out of 62 genes, 8.1%	26 out of 5417 background genes, 0.5%	0.002	0.00%
22616	DNA strand elongation	5 out of 62 genes, 8.1%	28 out of 5417 background genes, 0.5%	0.00294	0.00%
51097	negative regulation of helicase activity	3 out of 62 genes, 4.8%	5 out of 5417 background genes, 0.1%	0.00309	0.00%
6974	DNA damage response	12 out of 62 genes, 19.4%	247 out of 5417 background genes, 4.6%	0.00384	0.00%
32780	negative regulation of ATP-dependent activity	3 out of 62 genes, 4.8%	6 out of 5417 background genes, 0.1%	0.00613	0.00%
724	double-strand break repair via homologous recombination	6 out of 62 genes, 9.7%	54 out of 5417 background genes, 1.0%	0.0065	0.00%
6310	DNA recombination	8 out of 62 genes, 12.9%	112 out of 5417 background genes, 2.1%	0.00763	0.10%
725	recombinational repair	6 out of 62 genes, 9.7%	60 out of 5417 background genes, 1.1%	0.01196	0.18%
51095	regulation of helicase activity	3 out of 62 genes, 4.8%	8 out of 5417 background genes, 0.1%	0.01689	0.35%
6268	DNA unwinding involved in DNA replication	3 out of 62 genes, 4.8%	8 out of 5417 background genes, 0.1%	0.01689	0.33%
7049	cell cycle	15 out of 62 genes, 24.2%	439 out of 5417 background genes, 8.1%	0.01958	0.32%
6302	double-strand break repair	7 out of 62 genes, 11.3%	95 out of 5417 background genes, 1.8%	0.02012	0.31%
2E+06	mitotic DNA replication initiation	3 out of 62 genes, 4.8%	9 out of 5417 background genes, 0.2%	0.02512	0.30%
2E+06	regulation of DNA duplex unwinding	2 out of 62 genes, 3.2%	2 out of 5417 background genes, 0.0%	0.02836	1.00%
2E+06	negative regulation of DNA duplex unwinding	2 out of 62 genes, 3.2%	2 out of 5417 background genes, 0.0%	0.02836	0.97%
2E+06	regulation of DNA helicase activity	2 out of 62 genes, 3.2%	2 out of 5417 background genes, 0.0%	0.02836	0.93%
2E+06	negative regulation of DNA helicase activity	2 out of 62 genes, 3.2%	2 out of 5417 background genes, 0.0%	0.02836	0.90%
2E+06	cell cycle DNA replication initiation	3 out of 62 genes, 4.8%	10 out of 5417 background genes, 0.2%	0.0356	1.06%
2E+06	nuclear cell cycle DNA replication initiation	3 out of 62 genes, 4.8%	10 out of 5417 background genes, 0.2%	0.0356	1.03%
22402	cell cycle process	14 out of 62 genes, 22.6%	411 out of 5417 background genes, 7.6%	0.03661	1.00%
2E+06	mitotic DNA replication	4 out of 62 genes, 6.5%	26 out of 5417 background genes, 0.5%	0.04238	0.97%

Molecular function

GOID	GO_term	Cluster frequency	Background frequency	Corrected *p* -value	False discovery rate
17116	single-stranded DNA helicase activity	5 out of 62 genes, 8.1%	9 out of 5417 background genes, 0.2%	1.44E-06	0.00%
3688	DNA replication origin binding	7 out of 62 genes, 11.3%	29 out of 5417 background genes, 0.5%	1.65E-06	0.00%
2E+06	single-stranded 3'-5' DNA helicase activity	3 out of 62 genes, 4.8%	3 out of 5417 background genes, 0.1%	0.0001	0.00%
33679	3'-5' DNA/RNA helicase activity	3 out of 62 genes, 4.8%	3 out of 5417 background genes, 0.1%	0.0001	0.00%

9378	four-way junction helicase activity	3 out of 62 genes, 4.8%	5 out of 5417 background genes, 0.1%	0.00099	0.00%
3678	DNA helicase activity	5 out of 62 genes, 8.1%	32 out of 5417 background genes, 0.6%	0.00188	0.00%
3697	single-stranded DNA binding	6 out of 62 genes, 9.7%	61 out of 5417 background genes, 1.1%	0.00424	0.00%
33677	DNA/RNA helicase activity	3 out of 62 genes, 4.8%	8 out of 5417 background genes, 0.1%	0.00545	0.00%
140097	catalytic activity, acting on DNA	8 out of 62 genes, 12.9%	131 out of 5417 background genes, 2.4%	0.00756	0.00%
2E+06	sequence-specific double-stranded DNA binding	7 out of 62 genes, 11.3%	101 out of 5417 background genes, 1.9%	0.00958	0.60%
8094	ATP-dependent activity, acting on DNA	6 out of 62 genes, 9.7%	79 out of 5417 background genes, 1.5%	0.0181	0.91%
43138	3'-5' DNA helicase activity	3 out of 62 genes, 4.8%	13 out of 5417 background genes, 0.2%	0.02672	0.83%

Cellular component

GOID	GO_term	Cluster frequency	Background frequency	Corrected *p* -value	False discovery rate
42555	MCM complex	5 out of 62 genes, 8.1%	5 out of 5417 background genes, 0.1%	1.11E-08	0.00%
36387	pre-replicative complex	6 out of 62 genes, 9.7%	16 out of 5417 background genes, 0.3%	8.62E-07	0.00%
5656	nuclear pre-replicative complex	6 out of 62 genes, 9.7%	16 out of 5417 background genes, 0.3%	8.62E-07	0.00%
71162	CMG complex	5 out of 62 genes, 8.1%	10 out of 5417 background genes, 0.2%	2.69E-06	0.00%
31261	DNA replication preinitiation complex	6 out of 62 genes, 9.7%	21 out of 5417 background genes, 0.4%	5.58E-06	0.00%
31298	replication fork protection complex	6 out of 62 genes, 9.7%	21 out of 5417 background genes, 0.4%	5.58E-06	0.00%
5657	replication fork	8 out of 62 genes, 12.9%	53 out of 5417 background genes, 1.0%	7.34E-06	0.00%
43596	nuclear replication fork	7 out of 62 genes, 11.3%	40 out of 5417 background genes, 0.7%	1.69E-05	0.00%
97373	MCM core complex	3 out of 62 genes, 4.8%	3 out of 5417 background genes, 0.1%	9.56E-05	0.00%
5835	fatty acid synthase complex	2 out of 62 genes, 3.2%	2 out of 5417 background genes, 0.0%	0.00863	0.00%
228	nuclear chromosome	7 out of 62 genes, 11.3%	124 out of 5417 background genes, 2.3%	0.03221	1.82%

Significantly enriched gene groups in Farnesol treated cultures

Biological process

GOID	GO_term	Cluster frequency	Background frequency	Corrected *p* -value	False discovery rate
6355	regulation of DNA-templated transcription	45 out of 258 genes, 17.4%	437 out of 5417 background genes, 8.1%	0.0003	0.00%
2E+06	regulation of RNA biosynthetic process	45 out of 258 genes, 17.4%	438 out of 5417 background genes, 8.1%	0.00032	0.00%
51252	regulation of RNA metabolic process	47 out of 258 genes, 18.2%	485 out of 5417 background genes, 9.0%	0.00096	0.00%
10556	regulation of macromolecule biosynthetic process	51 out of 258 genes, 19.8%	570 out of 5417 background genes, 10.5%	0.0035	0.00%

31326	regulation of cellular biosynthetic process	51 out of 258 genes, 19.8%	588 out of 5417 background genes, 10.9%	0.00861	1.20%
9889	regulation of biosynthetic process	52 out of 258 genes, 20.2%	607 out of 5417 background genes, 11.2%	0.00989	1.00%
19219	regulation of nucleobase-containing compound metabolic process	49 out of 258 genes, 19.0%	562 out of 5417 background genes, 10.4%	0.01156	0.86%

Molecular function

GOID	GO_term	Cluster frequency	Background frequency	Corrected *p* -value	False discovery rate
3700	DNA-binding transcription factor activity	27 out of 258 genes, 10.5%	149 out of 5417 background genes, 2.8%	1.80E-07	0.00%
140110	transcription regulator activity	30 out of 258 genes, 11.6%	199 out of 5417 background genes, 3.7%	1.94E-06	0.00%
981	DNA-binding transcription factor activity, RNA polymerase II-specific	21 out of 258 genes, 8.1%	121 out of 5417 background genes, 2.2%	3.10E-05	0.00%

Cellular component

GOID	GO_term	Cluster frequency	Background frequency	Corrected *p* -value	False discovery rate
71944	cell periphery	33 out of 258 genes, 12.8%	353 out of 5417 background genes, 6.5%	0.01878	2.00%

The overlaps between Tyrosol and Farnesol responsive genes

Biological process

GOID	GO_term	Gene(s) annotated to the term
2E+06	regulation of RNA helicase activity	B9J08_005188:B9J08_005385

Molecular function

GOID	GO_term	Gene(s) annotated to the term
43024	ribosomal small subunit binding	B9J08_000442:B9J08_005385

Cellular component

GOID	GO_term	Gene(s) annotated to the term
2E+06	ferroxidase complex	B9J08_002108:B9J08_003002
33573	high-affinity iron permease complex	B9J08_002108:B9J08_003002

Significantly enriched gene groups in Tyrosol treated cultures		
**Biological process**		
GOID	GO_term	Gene(s) annotated to the term
2E+06	pre-replicative complex assembly involved in cell cycle DNA replication	B9J08_000087:B9J08_001105:B9J08_002424:B9J08_003467:B9J08_003937:B9J08_005476
36388	pre-replicative complex assembly	B9J08_000087:B9J08_001105:B9J08_002424:B9J08_003467:B9J08_003937:B9J08_005476
6267	pre-replicative complex assembly involved in nuclear cell cycle DNA replication	B9J08_000087:B9J08_001105:B9J08_002424:B9J08_003467:B9J08_003937:B9J08_005476
33260	nuclear DNA replication	B9J08_000087:B9J08_001105:B9J08_002424:B9J08_003467:B9J08_003937:B9J08_005422:B9J08_005476:B9J08_005515
44786	cell cycle DNA replication	B9J08_000087:B9J08_001105:B9J08_002424:B9J08_003467:B9J08_003937:B9J08_005422:B9J08_005476:B9J08_005515
6260	DNA replication	B9J08_000087:B9J08_001105:MRC1:B9J08_002424:B9J08_003338:B9J08_003467:B9J08_003937:B9J08_005422:B9J08_005476:B9J08_005515
32508	DNA duplex unwinding	B9J08_000087:B9J08_001105:B9J08_002424:B9J08_003467:B9J08_005476
6261	DNA-templated DNA replication	B9J08_000087:B9J08_001105:B9J08_002424:B9J08_003338:B9J08_003467:B9J08_003937:B9J08_005422:B9J08_005476:B9J08_005515
32392	DNA geometric change	B9J08_000087:B9J08_001105:B9J08_002424:B9J08_003467:B9J08_005476
6270	DNA replication initiation	B9J08_000087:B9J08_001105:B9J08_002424:B9J08_003467:B9J08_005476:B9J08_005515
6259	DNA metabolic process	B9J08_000087:B9J08_000425:B9J08_000447:B9J08_001105:MRC1:B9J08_002424:B9J08_003338:B9J08_003467:B9J08_003937:B9J08_003938:B9J08_005052:B9J08_005291:B9J08_005422:B9J08_005476:B9J08_005515
6281	DNA repair	B9J08_000087:B9J08_000447:B9J08_001105:MRC1:B9J08_002424:B9J08_003338:B9J08_003467:B9J08_003938:B9J08_005052:B9J08_005291:B9J08_005476:B9J08_005515
727	double-strand break repair via break-induced replication	B9J08_000087:B9J08_001105:B9J08_002424:B9J08_003467:B9J08_005476
6271	DNA strand elongation involved in DNA replication	B9J08_001105:B9J08_002424:B9J08_003338:B9J08_003467:B9J08_005476
71103	DNA conformation change	B9J08_000087:B9J08_001105:B9J08_002424:B9J08_003467:B9J08_005476
22616	DNA strand elongation	B9J08_001105:B9J08_002424:B9J08_003338:B9J08_003467:B9J08_005476
51097	negative regulation of helicase activity	B9J08_000087:B9J08_003467:B9J08_005476
6974	DNA damage response	B9J08_000087:B9J08_000447:B9J08_001105:MRC1:B9J08_002424:B9J08_003338:B9J08_003467:B9J08_003938:B9J08_005052:B9J08_005291:B9J08_005476:B9J08_005515
32780	negative regulation of ATP-dependent activity	B9J08_000087:B9J08_003467:B9J08_005476
724	double-strand break repair via homologous recombination	B9J08_000087:B9J08_001105:B9J08_002424:B9J08_003467:B9J08_003938:B9J08_005476

6310	DNA recombination	B9J08_000087:B9J08_000425:B9J08_001105:B9J08_002424:B9J08_003467:B9J08_003938:B9J08_005476:B9J08_005515
725	recombinational repair	B9J08_000087:B9J08_001105:B9J08_002424:B9J08_003467:B9J08_003938:B9J08_005476
51095	regulation of helicase activity	B9J08_000087:B9J08_003467:B9J08_005476
6268	DNA unwinding involved in DNA replication	B9J08_001105:B9J08_002424:B9J08_003467
7049	cell cycle	B9J08_000087:B9J08_000290:B9J08_000425:B9J08_000958:B9J08_001105:B9J08_001467:MRC1:B9J08_002424:B9J08_003338:B9J08_003467:B9J08_003937:B9J08_005205:B9J08_005422:B9J08_005476:B9J08_005515
6302	double-strand break repair	B9J08_000087:B9J08_001105:B9J08_002424:B9J08_003467:B9J08_003938:B9J08_005476:B9J08_005515
2E+06	mitotic DNA replication initiation	B9J08_003467:B9J08_005476:B9J08_005515
2E+06	regulation of DNA duplex unwinding	B9J08_003467:B9J08_005476
2E+06	negative regulation of DNA duplex unwinding	B9J08_003467:B9J08_005476
2E+06	regulation of DNA helicase activity	B9J08_003467:B9J08_005476
2E+06	negative regulation of DNA helicase activity	B9J08_003467:B9J08_005476
2E+06	cell cycle DNA replication initiation	B9J08_003467:B9J08_005476:B9J08_005515
2E+06	nuclear cell cycle DNA replication initiation	B9J08_003467:B9J08_005476:B9J08_005515
22402	cell cycle process	B9J08_000087:B9J08_000290:B9J08_000425:B9J08_000958:B9J08_001105:MRC1:B9J08_002424:B9J08_003338:B9J08_003467:B9J08_003937:B9J08_005205:B9J08_005422:B9J08_005476:B9J08_005515
2E+06	mitotic DNA replication	B9J08_001105:B9J08_003467:B9J08_005476:B9J08_005515

Molecular function

GOID	GO_term	Gene(s) annotated to the term
17116	single-stranded DNA helicase activity	B9J08_000087:B9J08_001105:B9J08_002424:B9J08_003467:B9J08_005476
3688	DNA replication origin binding	B9J08_000087:B9J08_001105:B9J08_002424:B9J08_003467:B9J08_003937:B9J08_005476:B9J08_005515
2E+06	single-stranded 3'-5' DNA helicase activity	B9J08_001105:B9J08_002424:B9J08_003467
33679	3'-5' DNA/RNA helicase activity	B9J08_001105:B9J08_002424:B9J08_003467
9378	four-way junction helicase activity	B9J08_001105:B9J08_002424:B9J08_003467
3678	DNA helicase activity	B9J08_000087:B9J08_001105:B9J08_002424:B9J08_003467:B9J08_005476
3697	single-stranded DNA binding	B9J08_000087:B9J08_001105:B9J08_002424:B9J08_003467:B9J08_005476:B9J08_005515
33677	DNA/RNA helicase activity	B9J08_001105:B9J08_002424:B9J08_003467
140097	catalytic activity, acting on DNA	B9J08_000087:B9J08_001105:B9J08_002424:B9J08_003338:B9J08_003467:B9J08_005052:B9J08_005476:B9J08_005515

2E+06	sequence-specific double-stranded DNA binding	B9J08_000087:B9J08_001105:B9J08_002424:B9J08_003467:B9J08_003937:B9J08_005476:B9J08_005515
8094	ATP-dependent activity, acting on DNA	B9J08_000087:B9J08_001105:B9J08_002424:B9J08_003338:B9J08_003467:B9J08_005476
43138	3'-5' DNA helicase activity	B9J08_001105:B9J08_002424:B9J08_003467

Cellular component

GOID	GO_term	Gene(s) annotated to the term
42555	MCM complex	B9J08_000087:B9J08_001105:B9J08_002424:B9J08_003467:B9J08_005476
36387	pre-replicative complex	B9J08_000087:B9J08_001105:B9J08_002424:B9J08_003467:B9J08_003937:B9J08_005476
5656	nuclear pre-replicative complex	B9J08_000087:B9J08_001105:B9J08_002424:B9J08_003467:B9J08_003937:B9J08_005476
71162	CMG complex	B9J08_000087:B9J08_001105:B9J08_002424:B9J08_003467:B9J08_005476
31261	DNA replication preinitiation complex	B9J08_000087:B9J08_001105:B9J08_002424:B9J08_003467:B9J08_003937:B9J08_005476
31298	replication fork protection complex	B9J08_000087:B9J08_001105:MRC1:B9J08_002424:B9J08_003467:B9J08_005476
5657	replication fork	B9J08_000087:B9J08_001105:MRC1:B9J08_002424:B9J08_003338:B9J08_003467:B9J08_005476:B9J08_005515
43596	nuclear replication fork	B9J08_000087:B9J08_001105:MRC1:B9J08_002424:B9J08_003467:B9J08_005476:B9J08_005515
97373	MCM core complex	B9J08_001105:B9J08_002424:B9J08_003467
5835	fatty acid synthase complex	B9J08_001296:B9J08_002420
228	nuclear chromosome	B9J08_000087:B9J08_001105:MRC1:B9J08_002424:B9J08_003467:B9J08_005476:B9J08_005515

Significantly enriched gene groups in Farnesol treated cultures

Biological process

GOID	GO_term	Gene(s) annotated to the term
6355	regulation of DNA-templated transcription	B9J08_000152:B9J08_000341:B9J08_000378:FLO8:B9J08_000592:B9J08_000752:B9J08_000772:B9J08_000869:B9J08_000984:B9J08_000993:B9J08_001265:B9J08_001285:B9J08_001371:B9J08_001642:RAD9:B9J08_002136:B9J08_002230:B9J08_002344:B9J08_002423:B9J08_002529:B9J08_002687:B9J08_002710:B9J08_002834:B9J08_002978:B9J08_003195:B9J08_003216:B9J08_003227:B9J08_003301:B9J08_003421:B9J08_003454:B9J08_003712:B9J08_003720:B9J08_003936:B9J08_003964:B9J08_004089:B9J08_004167:B9J08_004271:MRR1c:B9J08_004491:B9J08_004749:B9J08_005107:B9J08_005114:B9J08_005317:B9J08_005567:B9J08_005579

2E+06	regulation of RNA biosynthetic process	B9J08_000152:B9J08_000341:B9J08_000378:FLO8:B9J08_000592:B9J08_000752:B9J08_000772:B9J08_000869:B9J08_000984:B9J08_000993:B9J08_001265:B9J08_001285:B9J08_001371:B9J08_001642:RAD9:B9J08_002136:B9J08_002230:B9J08_002344:B9J08_002423:B9J08_002529:B9J08_002687:B9J08_002710:B9J08_002834:B9J08_002978:B9J08_003195:B9J08_003216:B9J08_003227:B9J08_003301:B9J08_003421:B9J08_003454:B9J08_003712:B9J08_003720:B9J08_003936:B9J08_003964:B9J08_004089:B9J08_004167:B9J08_004271:MRR1c:B9J08_004491:B9J08_004749:B9J08_005107:B9J08_005114:B9J08_005317:B9J08_005567:B9J08_005579
51252	regulation of RNA metabolic process	B9J08_000152:B9J08_000341:B9J08_000378:FLO8:B9J08_000592:B9J08_000752:B9J08_000772:B9J08_000869:B9J08_000984:B9J08_000993:B9J08_001265:B9J08_001285:B9J08_001371:B9J08_001439:B9J08_001642:RAD9:B9J08_002136:B9J08_002230:B9J08_002344:B9J08_002423:B9J08_002529:B9J08_002687:B9J08_002710:B9J08_002834:B9J08_002978:B9J08_003195:B9J08_003216:B9J08_003227:B9J08_003301:B9J08_003421:B9J08_003454:B9J08_003712:B9J08_003720:B9J08_003936:B9J08_003964:B9J08_004089:B9J08_004167:B9J08_004271:MRR1c:B9J08_004491:B9J08_004749:B9J08_005107:B9J08_005114:B9J08_005317:B9J08_005419:B9J08_005567:B9J08_005579
10556	regulation of macromolecule biosynthetic process	B9J08_000152:B9J08_000260:B9J08_000341:B9J08_000378:FLO8:B9J08_000592:B9J08_000752:B9J08_000772:B9J08_000862:B9J08_000869:B9J08_000984:B9J08_000993:B9J08_001265:B9J08_001285:B9J08_001371:B9J08_001642:RAD9:B9J08_002136:B9J08_002230:B9J08_002261:B9J08_002344:B9J08_002423:B9J08_002529:B9J08_002687:B9J08_002710:B9J08_002834:B9J08_002896:B9J08_002978:B9J08_003195:B9J08_003216:B9J08_003227:B9J08_003301:B9J08_003421:B9J08_003454:B9J08_003712:B9J08_003720:B9J08_003936:B9J08_003964:B9J08_004089:B9J08_004167:B9J08_004271:B9J08_004300:MRR1c:B9J08_004491:B9J08_004749:B9J08_005107:B9J08_005114:B9J08_005317:B9J08_005419:B9J08_005567:B9J08_005579
31326	regulation of cellular biosynthetic process	B9J08_000152:B9J08_000260:B9J08_000341:B9J08_000378:FLO8:B9J08_000592:B9J08_000752:B9J08_000772:B9J08_000862:B9J08_000869:B9J08_000984:B9J08_000993:B9J08_001265:B9J08_001275:B9J08_001285:B9J08_001371:B9J08_001642:RAD9:B9J08_002136:B9J08_002230:B9J08_002261:B9J08_002344:B9J08_002423:B9J08_002529:B9J08_002687:B9J08_002710:B9J08_002834:B9J08_002978:B9J08_003195:B9J08_003216:B9J08_003227:B9J08_003301:B9J08_003421:B9J08_003454:B9J08_003712:B9J08_003720:B9J08_003936:B9J08_003964:B9J08_004089:B9J08_004167:B9J08_004271:B9J08_004300:MRR1c:B9J08_004491:B9J08_004749:B9J08_005107:B9J08_005114:B9J08_005317:B9J08_005419:B9J08_005567:B9J08_005579
9889	regulation of biosynthetic process	B9J08_000152:B9J08_000260:B9J08_000341:B9J08_000378:FLO8:B9J08_000592:B9J08_000752:B9J08_000772:B9J08_000862:B9J08_000869:B9J08_000984:B9J08_000993:B9J08_001265:B9J08_001275:B9J08_001285:B9J08_001371:B9J08_001642:RAD9:B9J08_002136:B9J08_002230:B9J08_002261:B9J08_002344:B9J08_002423:B9J08_002529:B9J08_002687:B9J08_002710:B9J08_002834:B9J08_002896:B9J08_002978:B9J08_003195:B9J08_003216:B9J08_003227:B9J08_003301:B9J08_003421:B9J08_003454:B9J08_003712:B9J08_003720:B9J08_003936:B9J08_003964:B9J08_004089:B9J08_004167:B9J08_004271:B9J08_004300:MRR1c:B9J08_004491:B9J08_004749:B9J08_005107:B9J08_005114:B9J08_005317:B9J08_005419:B9J08_005567:B9J08_005579

19219	regulation of nucleobase-containing compound metabolic process	B9J08_000152:B9J08_000341:B9J08_000378:FLO8:B9J08_000592:B9J08_000752:B9J08_000772:B9J08_000862:B9J08_000869:B9J08_000984:B9J08_000993:B9J08_001265:B9J08_001285:B9J08_001371:B9J08_001439:B9J08_001642:B9J08_002078:RAD9:B9J08_002136:B9J08_002230:B9J08_002344:B9J08_002423:B9J08_002529:B9J08_002687:B9J08_002710:B9J08_002834:B9J08_002978:B9J08_003195:B9J08_003216:B9J08_003227:B9J08_003301:B9J08_003421:B9J08_003454:B9J08_003712:B9J08_003720:B9J08_003936:B9J08_003964:B9J08_004089:B9J08_004167:B9J08_004271:MRR1c:B9J08_004491:B9J08_004749:B9J08_005107:B9J08_005114:B9J08_005317:B9J08_005419:B9J08_005567:B9J08_005579

Molecular function

GOID	GO_term	Gene(s) annotated to the term
3700	DNA-binding transcription factor activity	B9J08_000341:FLO8:B9J08_000592:B9J08_000772:B9J08_000862:B9J08_000981:B9J08_001285:B9J08_001371:B9J08_002136:B9J08_002230:B9J08_002344:B9J08_002423:B9J08_002529:B9J08_002687:B9J08_002710:B9J08_002834:B9J08_003227:B9J08_003301:B9J08_003712:B9J08_003964:B9J08_004089:B9J08_004167:B9J08_004171:B9J08_004271:MRR1c:B9J08_005107:B9J08_005317
140110	transcription regulator activity	B9J08_000341:FLO8:B9J08_000592:B9J08_000772:B9J08_000862:B9J08_000981:B9J08_000993:B9J08_001265:B9J08_001285:B9J08_001371:B9J08_002136:B9J08_002230:B9J08_002344:B9J08_002423:B9J08_002529:B9J08_002687:B9J08_002710:B9J08_002834:B9J08_003227:B9J08_003301:B9J08_003712:B9J08_003964:B9J08_004089:B9J08_004167:B9J08_004171:B9J08_004271:MRR1c:B9J08_004749:B9J08_005107:B9J08_005317
981	DNA-binding transcription factor activity, RNA polymerase II-specific	FLO8:B9J08_000592:B9J08_000772:B9J08_000862:B9J08_001285:B9J08_001371:B9J08_002136:B9J08_002230:B9J08_002344:B9J08_002423:B9J08_002687:B9J08_002710:B9J08_003227:B9J08_003301:B9J08_003712:B9J08_003964:B9J08_004089:B9J08_004167:MRR1c:B9J08_005107:B9J08_005317

Cellular component

GOID	GO_term	Gene(s) annotated to the term
71944	cell periphery	B9J08_000092:B9J08_000220:B9J08_000228:B9J08_000517:B9J08_000822:B9J08_000984:B9J08_001082:B9J08_001275:B9J08_001834:B9J08_002109:B9J08_002110:B9J08_002167:B9J08_002261:B9J08_002789:B9J08_002855:B9J08_002877:B9J08_002895:B9J08_003120:B9J08_003387:B9J08_003623:B9J08_003703:B9J08_003957:B9J08_004259:B9J08_004286:B9J08_004339:B9J08_004383:B9J08_004775:B9J08_004786:B9J08_004935:B9J08_005075:B9J08_005148:B9J08_005346:B9J08_005380

Supplementary Table 3: Transcription data of selected gene groups.

Part 1: Genes involved in genetic control of *Candida auris* virulence.
Part 2: Genes involved in metabolism.
Part 3: Genes involved in ergosterol and fatty acid metabolic process.
Part 4: Genes involved in response to oxidative stress
Part 5: Genes involved in metal metabolism.

The systematic names, gene names and the features (putative molecular function or biological process) of the genes are given according to the Candida Genome Database (http://www.candidagenome.org).
Up- and down-regulated gene were defined as differentially expressed genes with < 0.05 corrected *p* value.
RNA-Seq data are presented as FC values, where FC is "fold change".
Up- and down-regulated genes are marked with red and blue colour.
Results of gene enrichment analysis (Fisher's exact test) are also enclosed.

Part 1: Selected genes involved in genetic control of *Candida auris* virulence.

C. albicans Gene ID	*C. albicans* Gene name	*C. auris* Gene ID	Tyrosol *vs.* Control
**Morphogenesis**			
CR_07890W	*EFG1*	B9J08_002834	-1.3736176
C1_07370C	*CPH1*	B9J08_003159	-1.0226402
C4_03570W	*HWP1*	B9J08_001409	1.0061085
C1_00780C	*HGC1*	B9J08_004946	-1.3376756
C1_13450W	*HYR1*	B9J08_004098	2.8838582
CR_07070C	*ALS3*	B9J08_004112	1.4342246
CR_09650W	*WAL1*	B9J08_000561	-1.5077319
C2_07930C	*VRP1*	B9J08_004190	-1.6436285
**Adhesion**			
C6_03700W	*ALS1*	B9J08_004112	1.4342246
C6_04380W	*ALS2*	B9J08_004112	1.4342246
CR_07070C	*ALS3*	B9J08_004112	1.4342246
C6_04130C	*ALS4*	B9J08_004113	2.5652137
C6_03690W	*ALS5*	B9J08_004112	1.4342246
C3_06190C	*ALS6*	B9J08_004112	1.4342246
C3_06320W	*ALS7*	B9J08_002582	1.4684114
C6_03710W	*ALS9*	B9J08_004498	1.8183211
C4_03570W	*HWP1*	B9J08_001409	1.0061085
CR_00610W	*IFF4*	B9J08_004098	2.8838582
C2_10030C	*MP65*	B9J08_003799	-1.2742085
**Invasion**			
C6_01990W	*PLB1*	B9J08_003486	1.0569062
C6_02000W	*PLB2*	B9J08_003486	1.0569062
CR_09690C	*PLB3*	B9J08_003486	1.0569062
CR_09690C	*PLB4*	B9J08_003486	1.0569062
C1_08230C	*PLB5*	B9J08_003621	-1.2078103
C7_03710C	*PLC1*	B9J08_003873	1.081123
C2_03040W	*PLC2*	B9J08_004962	-1.2170849
C1_11590W	*PLD1*	B9J08_001576	-1.0938803
C1_09580C	*LIP1*	B9J08_004173	-1.1973815
C1_09420W	*LIP2*	B9J08_004176	1.9867206
C1_09900W	*LIP3*	B9J08_004172	-1.1033318
C6_04490W	*LIP4*	B9J08_004176	1.9867206
C7_02830C	*LIP5*	B9J08_004173	-1.1973815
C1_09600C	*LIP6*	B9J08_004173	-1.1973815
CR_09220C	*LIP7*	B9J08_004176	1.9867206
C7_03300C	*LIP8*	B9J08_004156	-1.4247698
C7_02880C	*LIP9*	B9J08_004176	1.9867206
C1_09590C	*LIP10*	B9J08_004176	1.9867206
C6_03490C	*SAP1*	B9J08_001518	-1.0055044
CR_07800W	*SAP2*	B9J08_001518	-1.0055044
C3_05230W	*SAP3*	B9J08_001534	-1.086402
C6_03500C	*SAP4*	B9J08_001518	-1.0055044
C6_03030W	*SAP5*	B9J08_001518	-1.0055044

C. albicans Gene ID	*C. albicans* Gene name	*C. auris* Gene ID	Tyrosol *vs.* Control
**Invasion**			
C6_02710C	*SAP6*	B9J08_001518	-1.0055044
C1_04870W	*SAP7*	B9J08_001958	1.2643087
C3_02510C	*SAP8*	B9J08_001534	-1.086402
C3_03870C	*SAP9*	B9J08_001958	1.2643087
C4_04470W	*SAP10*	B9J08_001958	1.2643087
C1_13480W	*SSA1, HSP70*	B9J08_000483	3.2778795
**Biofilm- Adhesion and/or colonisation**			
C6_03700W	*ALS1*	B9J08_004112	1.4342246
CR_07070C	*ALS3*	B9J08_004112	1.4342246
C6_03690W	*ALS5*	B9J08_004112	1.4342246
C4_03570W	*HWP1*	B9J08_001409	1.0061085
C4_00450C	*PGA10*	B9J08_001951	-2.4017844
C1_04020C	*CSH1*	B9J08_003568	-1.4274902
C4_06820C	*CZF1*	B9J08_003772	-1.4646612
**Biofilm- Maturation**			
C4_03520C	*RBT1*	B9J08_001458	2.7568634
C4_03570W	*HWP1*	B9J08_001409	1.0061085
CR_06440C	*BCR1*	B9J08_003985	-2.703008
CR_07890W	*EFG1*	B9J08_002834	-1.3736176
C2_08490W	*DSE1*	B9J08_005070	-1.1610816
C3_04530C	*TEC1*	B9J08_003950	-1.1802102
C2_00140W	*NDT80*	B9J08_000388	-1.1619009
C1_13620W	*ROB1*	B9J08_003992	1.2451568
C1_05140W	*BRG1*	B9J08_002529	-1.1917892
C1_06280C	*UME6*	B9J08_000592	1.2121416
C6_00280W	*CPH2*	B9J08_001679	1.2626207
CR_07440W	*ACE2*	B9J08_000468	-1.2698236
C4_04850C	*ZAP1, CSR1*	B9J08_004167	1.0896479
C1_08220W	*CCR4*	B9J08_002888	-1.1735618
C1_02420C	*FKS1 GSC1*	B9J08_000964	-1.2258832
C1_04020C	*CSH1*	B9J08_003568	-1.4274902
C1_04140W	*IFD6*	B9J08_003568	-1.4274902
C1_10290W	*GCA1*	B9J08_004015	1.1147976
C1_10550C	*GCA2*	B9J08_004015	1.1147976
CR_02070C	*ADH5*	B9J08_001171	1.8958155
C4_01260W	*RLM1*	B9J08_000772	1.2674679
C4_02250C	*BGL2*	B9J08_001418	1.2365078
C4_04530C	*PHR1*	B9J08_000918	1.1166594
C1_02990C	*XOG1*	B9J08_003251	1.7149798
**Biofilm-Dispersion**			
C7_04230W	*NRG1*	B9J08_005429	-1.6527722
C2_09320C	*PES1*	B9J08_003031	-1.2259457
C1_06280C	*UME6*	B9J08_000592	1.2121416
C7_02030W	*HSP90*	B9J08_004918	1.2476825
**pH sensing**			
C4_04530C	*PHR1*	B9J08_000918	1.1166594

C. albicans Gene ID	*C. albicans* Gene name	*C. auris* Gene ID	Tyrosol *vs.* Control
**pH sensing**			
C1_00220W	*PHR2*	B9J08_000384	-1.0975454
C2_03400C	*DFG16*	B9J08_004279	-1.1130687
C5_01950C	*RIM21*	B9J08_004815	1.2285339
C1_09380W	*RIM20*	B9J08_003197	1.0208423
C1_14340C	*RIM101*	B9J08_003060	1.1180406
**Role in osmotic stress**			
C3_00320W	*GPP1 RHR21*	B9J08_004378	1.5416319
C6_02010C	*GPD2*	B9J08_003684	1.2907022
**Regulation**			
CR_00120C	*MKC1*	B9J08_002682	1.3188347
C2_03330C	*HOG1*	B9J08_004369	-1.1294794
C4_06480C	*CEK1*	B9J08_000458	-1.0060121
**Heat shock proteins**			
CR_08250C	*HSP104*	B9J08_000476	5.088587
C7_02030W	*HSP90*	B9J08_004918	1.2476825
C2_03390C	*HSP78*	B9J08_004285	2.9762232
C1_13480W	*SSA1, HSP70*	B9J08_000483	3.2778795
C1_04300C	*SSA2*	B9J08_003364	1.2080113
CR_06490C	*HSP60*	B9J08_003991	1.1319412
C1_09170W	*HSF1 CTA81*	B9J08_001120	2.2767076
C2_04010C	*HSP21*	B9J08_003627	5.0326653
C5_02080C	*HSP12*	B9J08_001940	1.1496731
C1_14090W	*HSP10*	B9J08_002967	1.2305636
**Metal acquisition**			
CR_07070C	*ALS3*	B9J08_004112	1.4342246
C2_08050C	*SIT1*	B9J08_002110	-1.2258482
C4_00130W	*RBT5*	B9J08_005545	-3.2082934
C4_00450C	*RBT51, PGA101*	B9J08_001951	-2.4017844
C7_00090C	*CSA1*	B9J08_001951	-2.4017844
C4_06920C	*CSA2*	B9J08_005545	-3.2082934
C4_00120W	*PGA7*	B9J08_004469	-1.5249431
C4_06980W	*PRA1*	B9J08_002992	-1.0490171
C3_03710W	*CCC1*	B9J08_002258	-1.2550259
C6_00790C	*CTR1*	B9J08_001856	-1.1337811

Number of up-regulated adhesion related genes:	3
Number of adhesion related genes:	6
Total number of up-regulated genes:	142
Total number of genes:	5417
p -value (Fisher's exact test):	**0.0003329**

Number of down-regulated adhesion related genes:	0
Number of adhesion related genes:	6
Total number of down-regulated genes:	108
Total number of genes:	5417
p -value (Fisher's exact test):	1

Part 2: Selected genes involved in metabolism.

C. albicans Gene ID	*C. albicans* Gene name	*C. auris* Gene ID	Tyrosol *vs.* Control
**Glycolysis**			
CR_06340C	*PGI1*	B9J08_000808	-1.1942815
C7_01800C	*PFK2*	B9J08_004928	1.015269
C5_04810W	*PFK1*	B9J08_004309	-1.2022558
C4_01750C	*FBA1*	B9J08_005239	1.1653594
C3_07440W	*TPI1*	B9J08_001875	1.0493213
C3_06870W	*TDH3*	B9J08_001227	1.1328342
C6_00750C	*PGK1*	B9J08_001860	1.0084623
C2_03270W	*GPM1*	B9J08_004375	-1.1443323
C1_04320W	*GPM2*	B9J08_003371	1.005147
C1_08500C	*ENO1*	B9J08_000274	1.0086505
C2_05460W	*CDC19*	B9J08_003632	-1.3835418
CR_04510W	*HXK2*	B9J08_002566	-1.1548653
CR_07150W	*GLK1*	B9J08_005226	1.4689955
CR_07490C	*GLK4*	B9J08_005226	1.4689955
C1_13140C	*TYE7*	B9J08_004171	1.0484998
C2_10590W	*GAL4*	B9J08_003585	1.029767
**TCA cycle**			
C4_03940C	*PYC2*	B9J08_005201	-1.1288494
CR_03500W	*CIT1*	B9J08_002652	1.0905842
CR_08210C	*ACO1*	B9J08_004914	1.0145637
CR_05790C	*ACO2*	B9J08_001702	1.5698259
C1_09630W	*IDH1*	B9J08_004224	1.100684
C2_03080W	*IDH2*	B9J08_002770	1.0999676
C3_00880W	*KGD1*	B9J08_002363	1.1483148
CR_07420W	*KGD2*	B9J08_000469	1.1698365
CR_06760C	*LSC2*	B9J08_005128	-1.076552
C1_01690C	*LSC1*	B9J08_000650	-1.1594344
C1_05260C	*SDH1*	B9J08_000507	1.0960982
CR_05180C	*SDH2*	B9J08_002689	1.463119
CR_04530W	*FUM11*	B9J08_001207	-1.020628
C3_07640C	*FUM12*	B9J08_001207	-1.020628
C4_01900C	*MDH1-1*	B9J08_000764	1.0614331
**Glyoxylate cycle**			
CR_05790C	*ACO2*	B9J08_001702	1.5698259
CR_08210C	*ACO1*	B9J08_004914	1.0145637
CR_03500W	*CIT1*	B9J08_002652	1.0905842
C1_04500W	*ICL1*	B9J08_003374	1.0096838
C1_09690W	*MLS1*	B9J08_002919	1.1493694
C2_10480W	*MDH1-3*	B9J08_003466	1.3145133

C. albicans Gene ID	*C. albicans* Gene name	*C. auris* Gene ID	Tyrosol *vs.* Control
**Fermentation/oxidative ethanol degradation**			
C4_01850C	*PDC12*	B9J08_002231	1.1762316
C4_06570C	*PDC11*	B9J08_002231	1.1762316
C3_02800W	*ADH4*	B9J08_004114	1.149658
CR_02070C	*ADH5*	B9J08_001171	1.8958155
C5_05050W	*ADH1*	B9J08_004331	1.1853011
C2_04470W	*ADH3*	B9J08_004600	1.0947262
C4_05130C	*ALD6*	B9J08_005580	-1.3745904
C2_02970C	*ALD5*	B9J08_003088	-1.0355662
**Pentose phosphate pathway (oxidative branch)**			
C1_08980C_A	*ZWF1*	B9J08_004550	1.449011
CR_06700C_A	*SOL3*	B9J08_005134	1.0869752
C1_13860C_A	*GND1*	B9J08_002350	1.0810323
**e pathway (non-oxidative branch)**			
C3_01480C	*RKI1*	B9J08_000186	1.14024
C7_00150W		B9J08_000895	-1.0840642
C1_08320W	*TKL1*	B9J08_003615	-1.1337144
CR_03720W	*TAL1*	B9J08_005228	1.3813702
**Pyruvate Dehydrogenase Complex**			
C4_04150C	*PDB1*	B9J08_005207	-1.0133338
C4_07110C	*PDA1*	B9J08_003785	-1.0905085
C7_01640W	*LAT1*	B9J08_004737	-1.4931657
C1_13830C	*PDX1*	B9J08_003041	-1.1597375
CR_07400C	*LPD1*	B9J08_000976	-1.1261091
**Gluconeogenesis**			
C6_01670W	*MAE1*	B9J08_005497	-1.0036356
C4_01900C	*MDH1-1*	B9J08_000764	1.0614331
CR_00200W	*PCK1*	B9J08_002669	2.0402205
C1_08500C	*ENO1*	B9J08_000274	1.0086505
C2_03270W	*GPM1*	B9J08_004375	-1.1443323
C1_04320W	*GPM2*	B9J08_003371	1.005147
C6_00750C	*PGK1*	B9J08_001860	1.0084623
C3_06870W	*TDH3*	B9J08_001227	1.1328342
C4_01750C	*FBA1*	B9J08_005239	1.1653594
C3_07830W	*FBP1*	B9J08_005163	1.1648254
CR_06340C	*PGI1*	B9J08_000808	-1.1942815
**Trehalose Metabolism**			
CR_05720W	*TPS1*	B9J08_001695	1.450636
C1_03380W	*TPS2*	B9J08_000775	1.1395866
C2_10690W	*TPS3*	B9J08_001873	1.5995309
CR_00560W	*NTH1*	B9J08_000695	-1.0512638
C1_06940C	*ATC1*	B9J08_003397	-1.7091043
CR_07150W	*GLK1*	B9J08_005226	1.4689955
CR_07490C	*GLK4*	B9J08_005226	1.4689955

C. albicans Gene ID	*C. albicans* Gene name	*C. auris* Gene ID	Tyrosol *vs.* Control
**Glycogen metabolism**			
Glycogen catabolism			
C7_00930W	*GPH1*	B9J08_003276	-1.180223
C4_05140C	*GDB1*	B9J08_005581	-1.109489
C3_01320C	*SGA1*	B9J08_000365	1.0799321
Glycogen biosynthesis			
C1_01360C		B9J08_000997	-1.9628319
CR_00780C	*GSY1*	B9J08_001025	-1.2592763
C3_06450W	*GLG2*	B9J08_004833	1.0663264
C6_03340C	*GLC3*	B9J08_002986	-1.1871773
**Glycerol metabolism**			
C2_10240W	*GPD1*	B9J08_003449	-1.0315937
C6_02010C	*GPD2*	B9J08_003684	1.2907022
CR_05220C	*GUT1*	B9J08_004380	-1.7087883
C4_06760W	*GUT2*	B9J08_001799	1.0545174
C3_00320W	*GPP1*	B9J08_004378	1.5416319
C1_09190C	*DAK2*	B9J08_001368	1.0291587
C3_07340W	*GCY1*	B9J08_003568	-1.4274902
**Maltose degradation**			
CR_10790W	*MAL2*	B9J08_001255	1.0675261
C5_00220W	*ROT2*	B9J08_001301	-1.0319616
C5_04940W		B9J08_001255	1.0675261
C4_05140C	*GDB1*	B9J08_005581	-1.109489
C5_04940W		B9J08_001255	1.0675261
C7_00930W	*GPH1*	B9J08_003276	-1.180223
C1_10290W	*GCA1*	B9J08_004015	1.1147976
C1_10550C	*GCA2*	B9J08_004015	1.1147976
**D-arabinose degradation**			
C6_00150W	*ARD*	B9J08_000370	1.2050122
**Galactose degradation**			
CR_04660C	*UGP1*	B9J08_001989	1.0681819
**Inositol metabolism**			
C2_04940C	*ITR1*	B9J08_000172	1.1221843
CR_10100C	*INO1*	B9J08_002762	1.4407591

Part 3: Selected genes of ergosterol and fatty acid metabolic process.

C. albicans Gene ID	*C. albicans* Gene name	*C. auris* Gene ID	Tyrosol *vs.* Control
**Ergosterol biosynthetic process**			
C7_00700W	*CYB5*	B9J08_004742	-1.0608058
C1_08590C	*ERG1*	B9J08_000261	-1.0774068
C1_00800C	*ERG2*	B9J08_004943	-1.3228819
C1_04770C	*ERG3*	B9J08_003737	1.0994216
C3_00760W	*ERG4*	B9J08_002852	-1.2178051
C7_02840C	*ERG5*	B9J08_002349	1.0492753
C3_02150C	*ERG6*	B9J08_005340	-1.4212567
C2_02460W	*ERG7*	B9J08_005007	-1.2489257
C4_01870C	*ERG8*	B9J08_001389	1.2505677
C2_08610W	*ERG9*	B9J08_004587	-1.1531794
C2_04310W	*ERG10*	B9J08_003730	-1.2498504
C5_00660C	*ERG11*	B9J08_001448	1.0080801
C1_09460W	*ERG12*	B9J08_005525	-1.1222936
CR_09160C	*ERG13*	B9J08_004500	-1.2948077
C2_04580W	*ERG20*	B9J08_003125	-1.1089733
C2_09400C	*ERG24*	B9J08_003026	-1.0811259
CR_02370W	*ERG25*	B9J08_000367	1.0710282
C4_06270C	*ERG26*	B9J08_000920	-1.1135476
CR_01140C	*ERG27*	B9J08_004245	-1.0273219
**Fatty acid metabolic process**			
**Fatty acid biosynthesis - initial steps**			
CR_00640W	*ACC1*	B9J08_001018	-1.3785995
CR_10620C		B9J08_004120	-1.2086971
C3_04830C	*FAS2*	B9J08_002420	-1.669173
C5_00190C	*FAS1*	B9J08_001296	-1.5640616
**Fatty acid elongation - saturated**			
C3_04830C	*FAS2*	B9J08_002420	-1.669173
C5_00190C	*FAS1*	B9J08_001296	-1.5640616
**Fatty acid oxidation pathway**			
C6_03560W	*POX1*	B9J08_001740	1.0433357
C3_01930W	*PXP2*	B9J08_001741	1.5743753
C3_01960C	*POX1-3*	B9J08_001740	1.0433357
CR_08670C		B9J08_004905	1.077007
CR_08690C	*ECI1*	B9J08_004905	1.077007
CR_10160W	*FAA4*	B9J08_002763	1.0462605
C7_04180W	*FAA2-3*	B9J08_004931	-1.411582
C6_00740W	*FAT1*	B9J08_001861	1.1524413
C3_02810C	*FAA21*	B9J08_002781	1.0179542
C3_05980C	*FAA2*	B9J08_004059	-1.0026207
C2_06070W	*FAA2-1*	B9J08_002781	1.0179542
CR_00150C	*POT1*	B9J08_001484	1.113928
C3_01460C	*FOX3*	B9J08_001484	1.113928
C2_00780W	*POT1-2*	B9J08_001484	1.113928

C. albicans Gene ID	*C. albicans* Gene name	*C. auris* Gene ID	Tyrosol *vs.* Control
**Very long chain fatty acid biosynthesis**			
C1_01050C		B9J08_002971	1.1316886
C2_03230C	*FEN12*	B9J08_000511	-1.1277835
C3_05280C		B9J08_004437	-1.3913394
C1_12790C	*FEN1*	B9J08_003942	-1.1999226
CR_06070W		B9J08_002501	-1.0309244
**Unsaturated fatty acid biosynthetic process**			
C6_01110W	*FAD2*	B9J08_004590	-1.3126886
C1_13070C	*FAD3*	B9J08_004149	-1.4519187
C1_08360C	*OLE1*	B9J08_003612	-1.095344
C2_07090C	*OLE2*	B9J08_002798	1.1236199

Part 4: Genes involved in response to oxidative stress

C. albicans Gene ID	*C. albicans* Gene name	*C. auris* Gene ID	Tyrosol *vs.* Control
**Catalases, SODs, peroxidases, reductases, glutaredoxin, thioredoxin**			
C1_06810W	*CAT1*	B9J08_002298	1.5014946
C3_02480C	*CCP1*	B9J08_002366	1.7536365
C1_07180W	*CCS1*	B9J08_003869	1.1361938
C4_02320C	*SOD1*	B9J08_001381	1.3473425
C1_01520C	*SOD2*	B9J08_000528	1.1969761
C7_00110W	*SOD3*	B9J08_000528	1.1969761
C2_00660C	*SOD4*	B9J08_001292	-3.9931827
C2_00680C	*SOD5*	B9J08_001292	-3.9931827
C2_00240C	*SOD6*	B9J08_001640	1.0118377
C1_07880C	*GCS1*	B9J08_001605	-1.063427
C5_01520C	*GLR1*	B9J08_003819	-1.1484988
C6_00850W		B9J08_003442	1.3998983
C6_00840W	*GPX2*	B9J08_003442	1.3998983
C1_07350C	*GPX3*	B9J08_001814	1.3990368
C1_07630W		B9J08_005432	1.0686156
C4_02990C	*GST2*	B9J08_001170	-1.0554951
C3_00480C	*DOT5*	B9J08_004003	-1.0191329
C4_02710C	*GRX3*	B9J08_002026	-1.0255444
C5_01560C		B9J08_002920	1.0140673
C1_00490C	*TTR1*	B9J08_002920	1.0140673
C5_02710W	*TRR1*	B9J08_001090	1.2439033
CR_10350C	*TRX1*	B9J08_000543	1.3078516
C7_02810W	*PRX1*	B9J08_003437	1.3348007
C3_06180C	*TSA1*	B9J08_000645	1.2439436
C3_06330W	*TSA1B*	B9J08_000645	1.2439436
C6_00860W	*GPX1*	B9J08_003442	1.3998983
C4_02410C	*AHP1*	B9J08_002143	1.4402039
C2_05060C		B9J08_000169	2.4832706
**Quinone-dependent antioxidant system**			
CR_05380C	*YCP4*	B9J08_004840	1.6764432
C2_06870C	*PST1*	B9J08_004839	1.2461262
C2_08640C	*PST2*	B9J08_004839	1.2461262
CR_05390W	*PST3*	B9J08_004839	1.2461262
C2_07070W		B9J08_002479	1.5277524
**Detoxification**			
C3_05220W	*CDR1*	B9J08_000164	1.1239167
C3_04890W	*CDR2*	B9J08_000164	1.1239167
C6_03170C	*MDR1*	B9J08_003981	-1.7542409
C5_01840C	*TAC1*	B9J08_004819	1.0822847
C5_01850C	*ZNC1*	B9J08_004820	1.147646

C. albicans Gene ID	*C. albicans* Gene name	*C. auris* Gene ID	Tyrosol *vs.* Control
**Chaperon and related proteins**			
CR_04200W	*YDJ1*	B9J08_005368	-1.082465
C5_02110W		B9J08_001940	1.1496731
C2_04010C	*HSP21*	B9J08_003627	5.0326653
CR_05720W	*TPS1*	B9J08_001695	1.450636
C1_03380W	*TPS2*	B9J08_000775	1.1395866
**Regulation**			
C3_02220W	*CAP1*	B9J08_005344	1.4829279
C6_03580W	*CCZ1*	B9J08_003897	1.014944
C6_01070C	*CIP1*	B9J08_003024	2.491048
C2_07170C	*AFT2*	B9J08_002785	1.0630134
C3_04550C	*CMK1*	B9J08_000285	1.2639778
C2_10260C	*CMK2*	B9J08_003453	1.2863213
C4_01390W	*HAP3*	B9J08_004457	-1.1178128
CR_04290W	*HAP31*	B9J08_005483	1.0105096
C4_05110C	*HAP41*	B9J08_005579	1.0261477
C2_01700C	*HAP42*	B9J08_000152	1.3127034
C1_11210C	*HAP43*	B9J08_005298	-1.3003688
C5_00940C	*HAP5*	B9J08_002881	-1.0988716
C2_03330C	*HOG1*	B9J08_004369	-1.1294794
C1_08940C	*MSN4*	B9J08_002840	2.2408834
C7_00740W	*RIM15*	B9J08_003281	-1.0892241
C1_13960W	*GND1*	B9J08_002350	1.0810323
C2_03770C	*STE11*	B9J08_005331	-1.3840309
C2_03940C	*SCH9*	B9J08_000729	-1.1993895
C7_03220C	*ZCF29*	B9J08_002687	-1.2869884
CR_00120C	*MKC1*	B9J08_002682	1.3188347
C2_07130C	*RCK2*	B9J08_002792	1.2595477
C3_04860W	*SFP1*	B9J08_004443	-1.141492
C1_04380W	*SIT4*	B9J08_003383	-1.0513097
C4_05030C	*SSK2*	B9J08_005216	-1.0917753
C1_01200W	*STB5*	B9J08_004303	-1.1349069
C1_13930W	*ACT1*	B9J08_000486	1.0729159
C5_00240W	*SKN7*	B9J08_001300	-1.107481
C2_04260W	*SSN3*	B9J08_003736	1.062943
C2_02100W	*SNF2*	B9J08_001192	-1.2273847
C1_09140C	*SSU81*	B9J08_001931	1.0283786
CR_06420W	*PPZ1*	B9J08_003983	-1.0364577
C2_04980C	*CKA2*	B9J08_005003	1.025332
C2_10190C	*OCA1*	B9J08_003444	1.0104935
C2_10210C	*RAS1*	B9J08_003446	-1.1184504
**Other functions**			
C3_07340W	*GCY1*	B9J08_003568	-1.4274902
CR_00860C	*TMA19*	B9J08_002152	1.0055622
C6_03710W	*ALS9*	B9J08_004498	1.8183211
C2_03320W	*CHK1*	B9J08_001959	1.0068916
C1_03330C		B9J08_000779	1.3281585
CR_08990C	*SLP3*	B9J08_000987	1.2642839

C. albicans Gene ID	*C. albicans* Gene name	*C. auris* Gene ID	Tyrosol *vs.* Control
C3_01630W	*TFP1*	B9J08_001893	-1.040548
C1_11480W	*PHO84*	B9J08_001464	4.4935703
CR_09300C	*SCO1*	B9J08_004512	1.4045539
C7_02490W	*PEX1*	B9J08_005464	-1.1843531
C4_06430C		B9J08_000924	1.1215605
C1_04080W	*MAD2*	B9J08_005136	-1.0833098
CR_08960C	*MPS1*	B9J08_004652	-1.3551633
C2_05860C		B9J08_003179	-2.1354358
C6_02730W	*SAC6*	B9J08_001432	1.1464717
C5_05280C	*SDH4*	B9J08_003977	-1.1756991
C3_01300C	*NCE103*	B9J08_000363	1.1891319
C4_04640C	*ATG1*	B9J08_003355	1.709713
C2_00140W	*NDT80*	B9J08_000388	-1.1619009
C2_08100W		B9J08_000850	-1.1069446
CR_03010C	*ERV1*	B9J08_000344	1.0780791
C3_07010W	*POS5*	B9J08_004748	1.492941
C1_11880W		B9J08_001568	-1.0556259
C3_01850W		B9J08_001744	-1.2413427
C5_04530W		B9J08_004396	-1.162353
CR_08700C	*ARF1*	B9J08_002276	-1.1051639
CR_09790W	*ALO1*	B9J08_004501	1.0250709
C1_13860C	*IRA2*	B9J08_003924	-1.1654112
C1_08980C	*ZWF1*	B9J08_004550	1.449011
C3_03470W		B9J08_002737	1.4287643
C7_03350C		B9J08_000640	1.1030357
C3_06860C		B9J08_004583	1.2455956
C5_02930C	*GRE3*	B9J08_000812	-1.1602207
C6_02040W	*MCR1*	B9J08_003681	1.1392746
C1_11660W	*GAD1*	B9J08_001591	1.198617
CR_10250C	*FDH3*	B9J08_002769	1.2793105
C2_00960C	*MXR1*	B9J08_003339	1.0572593
C6_02800W		B9J08_001438	1.0632819
C1_01040W		B9J08_002972	1.248656
C4_00180W	*ECM7*	B9J08_003515	1.0754194
C4_04340C	*YFH1*	B9J08_004189	-1.1008555
CR_07790C	*YHB1*	B9J08_002691	1.5811614
C5_00810C		B9J08_002366	1.7536365
C1_00340W	*HBR1*	B9J08_004153	-1.0708416
C1_00350C	*HMX1*	B9J08_004152	-1.827736
C1_04670W	*HAT1*	B9J08_002458	-1.1090385
C6_04540C	*HAT2*	B9J08_005442	-1.2424723
C1_05270C		B9J08_003711	-1.2092807
C1_11160C		B9J08_005305	1.0242878
C4_05740C		B9J08_004875	-1.2487705
CR_01550C		B9J08_003506	1.0307716
CR_04300W		B9J08_005481	-1.356398
C3_02000W	*RAD50*	B9J08_004166	-1.1412182

C. albicans Gene ID	*C. albicans* Gene name	*C. auris* Gene ID	Tyrosol *vs.* Control
C6_00510C	*RAD52*	B9J08_000514	1.013384
C7_01340W	*MRE11*	B9J08_005411	1.0300651
C5_00500W	*NAM7*	B9J08_000442	-1.6569324
C7_04140C		B9J08_004920	1.0047433
C7_02480W		B9J08_005465	1.0378574
C1_10930C	*UBA4*	B9J08_000242	1.1142635
CR_06530W		B9J08_003987	1.0135671
C2_00840W		B9J08_003329	-1.0505798
C5_04420W		B9J08_004364	1.0419273
C3_02540C	*OXR1*	B9J08_000410	1.1603578
CR_05610C	*SRR1*	B9J08_005114	-1.0764557
C1_00400W	*SVF1*	B9J08_000654	-1.1319709
C1_09870W	*HCM1*	B9J08_003546	-1.5731667
CR_02650C	*DRE2*	B9J08_004088	1.3812044
C2_06720W	*GRE2*	B9J08_004828	4.7255926
CR_10650W	*LTV1*	B9J08_004117	-1.0368023
C1_06730W	*RAC1*	B9J08_003599	-1.0130875
C4_02030W	*RFX2*	B9J08_005242	1.0743564
C1_13930W	*SSK1*	B9J08_005450	-1.0901003
C1_13960W	*YBP1*	B9J08_004642	1.0402484
C3_07460W		B9J08_003576	1.0138901
C4_00180W	*ECM7*	B9J08_003515	1.0754194
C4_04640C	*GOA1*	B9J08_004211	1.0689294
CR_05980W		B9J08_000658	1.0729692
CR_08250C		B9J08_000476	5.088587

Number of up-regulated antioxidative defence related genes:	6
Number of antioxidative defence related genes:	21
Total number of up-regulated genes:	142
Total number of genes:	5417
p -value (Fisher's exact test):	**1.15E-05**

Number of down-regulated antioxidative defence related genes:	2
Number of antioxidative defence related genes:	21
Total number of down-regulated genes:	108
Total number of genes:	5417
p -value (Fisher's exact test):	0.06467

Part 5: Selected genes involved in iron metabolism

C. albicans Gene ID	*C. albicans* Gene name	*C. auris* Gene ID	Tyrosol *vs.* Control
**Ferric reductases**			
C4_05770C	*CFL1*	B9J08_004886	-2.617115
C4_05780C	*CFL2*	B9J08_004886	-2.617115
C4_05840W	*FRE3*	B9J08_001450	-2.0939221
C5_01380W	*CFL5*	B9J08_004886	-2.617115
C5_01360W	*CFL4*	B9J08_001450	-2.0939221
CR_07300W		B9J08_004886	-2.617115
CR_06670W	*CFL11*	B9J08_001450	-2.0939221
C2_05070W	*FRE9*	B9J08_000168	-2.436293
C4_04320W	*FRE10*	B9J08_000701	-1.9698305
C4_00110C	*FRP1*	B9J08_004468	-1.6260549
C7_00100W	*FRP2*	B9J08_004468	-1.6260549
CR_06870C		B9J08_004886	-2.617115
CR_07290W	*FRE7*	B9J08_004886	-2.617115
C2_03530W		B9J08_003455	1.1612761
C1_09780C		B9J08_004052	-1.0842334
**Multicopper oxidases**			
C6_00480C	*FET31*	B9J08_000517	-1.5103251
C6_00460C	*FET3*	B9J08_000517	-1.5103251
C5_00460C	*FET33*	B9J08_000517	-1.5103251
C6_00440C	*FET34*	B9J08_000517	-1.5103251
C6_00470C	*FET99*	B9J08_000517	-1.5103251
C5_03020W	*CCC2*	B9J08_002491	-1.0533291
**Iron permeases**			
C1_14130W	*FTR1*	B9J08_002108	-2.1300478
C1_14220C	*FTR2*	B9J08_002108	-2.1300478
C1_09400C	*FTH1*	B9J08_000170	1.1034563
CR_01270C	*FTH2*	B9J08_002464	-1.0442616
**Siderophore uptake**			
C2_08050C	*SIT1*	B9J08_002110	-1.2258482
**Hemoglobin/ Hem uptake**			
C4_00130W	*RBT5*	B9J08_005545	-3.2082934
C7_00090C	*CSA1*	B9J08_001951	-2.4017844
C4_06920C	*CSA2*	B9J08_005545	-3.2082934
C4_00120W	*PGA7*	B9J08_004469	-1.5249431
C4_00450C	*PGA10*	B9J08_001951	-2.4017844
C1_00350C	*HMX1*	B9J08_004152	-1.827736
**Regulation**			
CR_02190C	*SEF1*	B9J08_001107	-1.4907662
C1_10020W	*SFU1*	B9J08_000341	-1.208257
C2_07170C	*AFT2*	B9J08_002785	1.0630134
C7_00510W	*MAC1*	B9J08_001121	-1.0481342
C1_11210C	*HAP43*	B9J08_005298	-1.3003688

C. albicans Gene ID	*C. albicans* Gene name	*C. auris* Gene ID	Tyrosol *vs.* Control
**Intracellular homeostasis**			
C2_08070C	*MRS4*	B9J08_005181	-1.0741782
C3_03710W	*CCC1*	B9J08_002258	-1.2550259
C2_00580C	*SMF3*	B9J08_002199	1.0560555
C6_04210C	*ATM1*	B9J08_002232	-1.1504225
C6_00790C	*CTR1*	B9J08_001856	-1.1337811
C1_10720C	*MNN2*	B9J08_001935	-1.1136754
C2_07160W	*SMF12*	B9J08_002789	-1.05431
**Copper homeostasis and uptake**			
C1_09250W	*CRP1*	B9J08_002491	-1.0533291
C1_07180W	*CCS1*	B9J08_003869	1.1361938
C7_00510W	*MAC1*	B9J08_001121	-1.0481342
C6_00790C	*CTR1*	B9J08_001856	-1.1337811
C5_03020W	*CCC2*	B9J08_002491	-1.0533291
**Zinc homeostasis and uptake**			
C4_06970C	*ZRT1*	B9J08_000003	1.0256234
C2_02590W	*ZRT2*	B9J08_003657	-1.2046044
C4_06980W	*PRA1*	B9J08_002992	-1.0490171
C2_02200W	*ZRC1*	B9J08_001183	1.1697975
C2_02180W		B9J08_001185	1.1956779
C4_04850C	*CSR1*	B9J08_004167	1.0896479
**Manganase homeostasis and uptake**			
C7_00320C	*PMR1*	B9J08_000837	-1.186411
C3_03710W	*CCC1*	B9J08_002258	-1.2550259
C2_07160W	*SMF1*	B9J08_002789	-1.05431
C1_13840W		B9J08_003040	1.042415

Number of up-regulated iron metabolism related genes:	0
Number of iron metabolism related genes:	29
Total number of up-regulated genes:	142
Total number of genes:	5417
p -value (Fisher's exact test):	1

Number of down-regulated iron metabolism related genes:	6
Number of iron metabolism related genes:	29
Total number of down-regulated genes:	108
Total number of genes:	5417
p -value (Fisher's exact test):	**1.79E-05**

Supplementary Table 4. Results of RT-qPCR experiments

Gene ID	Gene name	Description	Tyrosol treated *vs* Untreated control	
			RNA-Seq (FC; mean)	RT-qPCR (ΔΔCP; mean ± S.D.)
B9J08_004098	*HYR1*	GPI-anchored hyphal cell wall protein	2.88	1.65± 0.12
B9J08_004176	*LIP4*	Secreted lipase	1.99	1.35± 0.06
B9J08_001458	*RBT1*	Cell wall protein with similarity to Hwp1	2.76	2.02± 0.31
B9J08_005429	*NRG1*	Transcription factor/repressor	-1.65	-1.34± 0.77
B9J08_003627	*HSP21*	Small heat shock protein	5.03	1.24± 0.54
B9J08_004309	*PFK1*	Phosphofructokinase alpha subinit	-1.20	-1.52± 0.94
B9J08_002231	*PDC11*	Pyruvate decarboxylase	1.18	0.61± 0.37
B9J08_004331	*ADH1*	Alcohol dehydrogenase	1.19	0.41± 0.33
B9J08_000261	*ERG1*	Squalene epoxidase	-1.08	-0.45± 0.37
B9J08_002420	*FAS2*	Alpha subunit of fatty-acid synthase	-1.67	-0.87± 0.31
B9J08_001296	*FAS1*	Beta subunit of fatty-acid synthase	-1.56	-0.98± 0.56
B9J08_002298	*CAT1*	Catalase	1.50	0.94± 0.07
B9J08_001292	*SOD4*	Cu-containing superoxide dismutase	-3.99	-1.575± 0.39
B9J08_000164	*CDR1*	Multidrug transporter of ABC superfamily	1.12	0.84± 0.74
B9J08_003024	*CIP1*	Possible oxidoreductase	2.49	1.55± 0.27
B9J08_002108	*FTR1*	High-affinity iron permease	-2.13	-1.44± 0.14

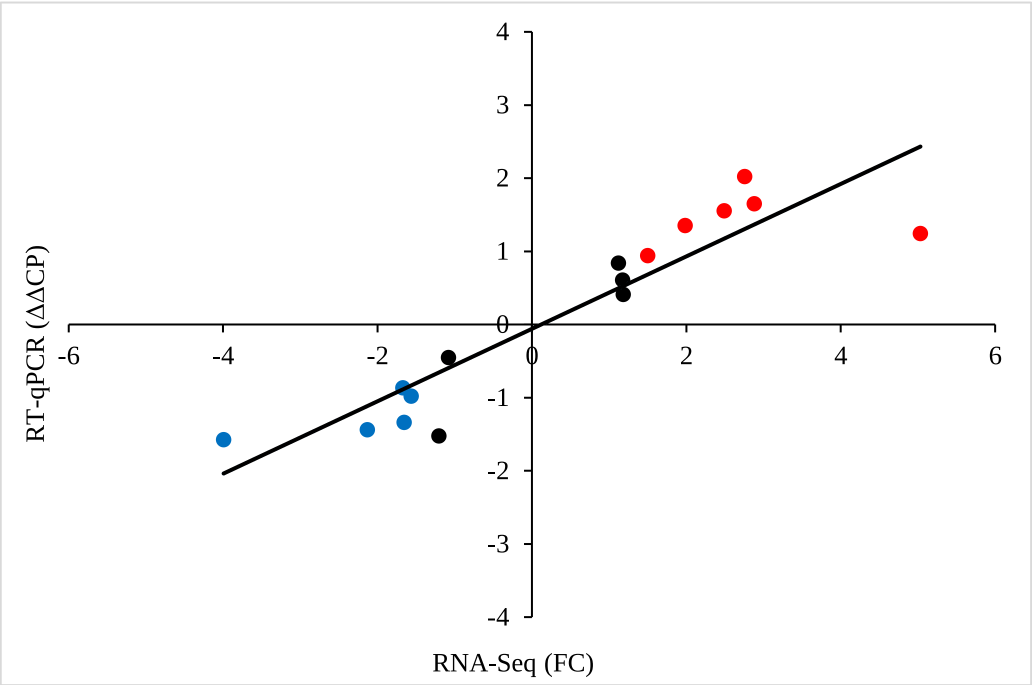

RNA-Seq data are presented as FC values, whereby FC is short for “fold-change”.
Relative transcription levels (ΔΔCP) were quantified with ΔΔCP = ΔCPcontrol –ΔCPtreated. CP values stand for the qRT-PCR cycle numbers of
RT-qPCR data are presented as mean ± SD calculated from three independent biological replicates.

Significantly higher or lower than zero ΔΔCP values (up-regulated or down-regulated gene) are marked with red and blue colors, respectively (Student’s *t* -test, p <0.05, n = 3). Black color indicates the non-significant values.

The diagram at the end of the table demonstrates the correlation between RT-qPCR and RNA-Seq data.
